# Probiotics restore enteric HDL3 secretion and improve prognosis in patients with end‐stage renal disease

**DOI:** 10.1002/imt2.70062

**Published:** 2025-07-01

**Authors:** Xiaoxue Liu, Yuan Huang, Yixuan Li, Juan Chen, Xifan Wang, Xiaobin Wang, Liang Zhao, Yongting Luo, Peng An, Liwei Zhang, Chengying Zhang, Weijing Bian, Xingen Lei, Xiang Gao, Yinghua Liu, Yanling Hao, Huiyuan Guo, Xiaoxu Zhang, Pengjie Wang, Ran Wang, Hao Zhang, Bing Fang, Xiaolin Zhang, Longjiao Wang, Qinglu Qiu, Yuchan Zhang, Jingyi Qi, Songtao Yang, Yulong Yin, Fazheng Ren, Xiaoyu Wang

**Affiliations:** ^1^ Key Laboratory of Precision Nutrition and Food Quality, Department of Nutrition and Health China Agricultural University Beijing China; ^2^ Cardiac Surgery Centre, Fuwai Hospital, National Center for Cardiovascular Diseases, Chinese Academy of Medical Sciences Peking Union Medical College Beijing China; ^3^ Department of Obstetrics and Gynecology Columbia University New York USA; ^4^ Division of General Pediatrics & Adolescent Medicine, Department of Pediatrics Johns Hopkins University School of Medicine Baltimore MD USA; ^5^ College of Food Science & Nutritional Engineering China Agricultural University Beijing China; ^6^ Department of General Practice The Third Medical Center of Chinese PLA General Hospital (Total Hospital of the Chinese People's Armed Police Force) Beijing China; ^7^ Department of Nephrology, Beijing Anzhen Hospital Capital Medical University Beijing China; ^8^ Department of Animal Science Cornell University, Ithaca New York USA; ^9^ Department of Nutrition and Food Hygiene, School of Public Health, Institute of Nutrition Fudan University Shanghai China; ^10^ Department of Nutrition The First Medical Center of Chinese PLA General Hospital Beijing China; ^11^ Food Laboratory of Zhongyuan Luohe China; ^12^ China Aerospace Science & Industry Corporation 731 Hospital Beijing China; ^13^ Yuelushan Laboratory Changsha China; ^14^ Key Laboratory of Livestock and Poultry Resources (Pig) Evaluation and Utilization, Ministry of Agriculture and Rural Affairs, College of Animal Science and Technology Hunan Agricultural University Changsha China; ^15^ Institute of Subtropical Agriculture Chinese Academy of Sciences Changsha China

## Abstract

Randomized double‐blind trials have shown that probiotic mixtures significantly increase high‐density lipoprotein (HDL) levels and reduce the risk of cardiovascular disease mortality in end‐stage renal disease (ESRD) patients. Meta‐analysis with prospective cohort studies further confirms that elevated HDL is a protective factor for ESRD outcomes. In severe renal injury models, including 5/6 nephrectomy and apolipoprotein E‐deficient (*ApoE^−/−^*) mice, probiotics restored cardiac function, mirroring the cardioprotective effects seen in humans. Mechanistic studies indicate that probiotics enhance intestinal HDL3 production through the insulin‐mediated SP1(P)‐CYP27A‐LXRα/β‐ABCA1 pathway, thereby maintaining HDL metabolic homeostasis. This study reveals a novel link between probiotic intervention and host cholesterol metabolism, offering a previously unexplored strategy for reducing cardiovascular risk in ESRD patients.

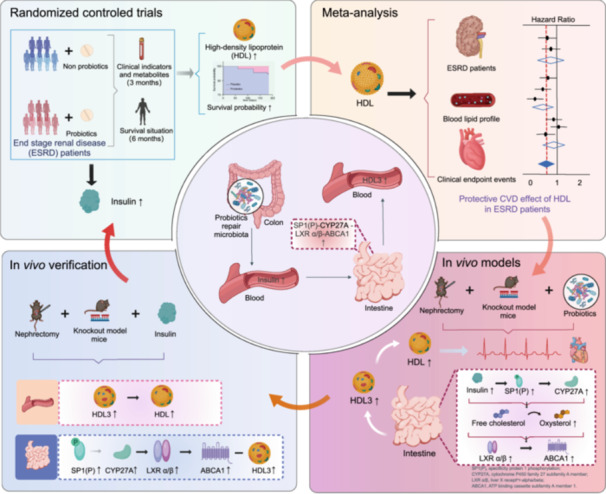

To the Editor,

End‐stage renal disease (ESRD), the terminal phase of chronic kidney disease, is associated with a disproportionately high risk of cardiovascular mortality [1, 2]. Conventional therapies like statins show limited efficacy in this population due to a distinct form of dyslipidemia compared to the general cardiovascular disease (CVD) population [3, 4]. Notably, ESRD patients often exhibit reduced high‐density lipoprotein (HDL) [5], a key mediator of cardiovascular protection through reverse cholesterol transport, anti‐inflammatory, and endothelial repair mechanisms [6, 7]. While preclinical studies suggest probiotics may modulate HDL metabolism [8], robust clinical evidence on their role in mitigating cardiovascular risk in ESRD remains scarce. To address this gap, the present study integrates meta‐analysis, randomized controlled clinical trials, and experimental mouse models to provide theoretical and clinical evidence supporting probiotic therapy for elevating HDL and improving cardiovascular health in patients with ESRD.

In our randomized controlled trial (NCT03010735), we evaluated a specific mixed probiotic formulation containing *Bifidobacterium animalis subsp. lactis* A6, *Lactobacillus rhamnosus* GG, *Lactobacillus acidophilus* A5, and *Lactobacillus paracasei* L9 in a 2:1:1:1 ratio—in 150 patients with ESRD. Following 6 months of intervention, the probiotic group exhibited a significant increase in HDL, primarily due to enhanced intestinal secretion of HDL3. This improvement was associated with a slowing of CVD progression and a reduced risk of mortality compared to the placebo group. Supporting these findings, a meta‐analysis for prospective cohorts further confirmed HDL normalization as a valuable prognostic marker in ESRD. Mechanistically, studies in 5/6 nephrectomized apolipoprotein E‐deficient (*ApoE⁻/⁻*) mice demonstrated that probiotics promote intestinal HDL3 biosynthesis through the insulin‐SP1(P)‐CYP27A‐LXRα/β‐ABCA1 pathway, leading to the attenuation of arteriosclerosis and myocardial injury, likely mediated by gut microbiota modulation.

These findings address a critical gap in the management of ESRD. In contrast to statins, which have limited success in reducing cardiovascular events despite lowering low‐density lipoprotein (LDL) [3, 4], the modulation of HDL through probiotic intervention presents a novel therapeutic strategy. The established safety profile of probiotics, characterized by minimal adverse effects in long‐term studies, further supports their potential for clinical translation. Furthermore, this study establishes a crucial link between gut‐derived HDL3 and systemic cardiovascular protection, providing valuable insights into the complex metabolic crosstalk between the microbiota and the host. Future research should focus on elucidating strain‐specific effects and optimizing dosage regimens to maximize therapeutic benefits. Ultimately, our work highlights the promising role of probiotic interventions in transforming cardiovascular care for patients with ESRD, effectively bridging mechanistic understanding with translational application.

## RESULTS AND DISCUSSION

### Probiotic intervention restores HDL levels and mitigates cardiovascular risk in ESRD patients through multi‐level evidence

**FIGURE 1:**
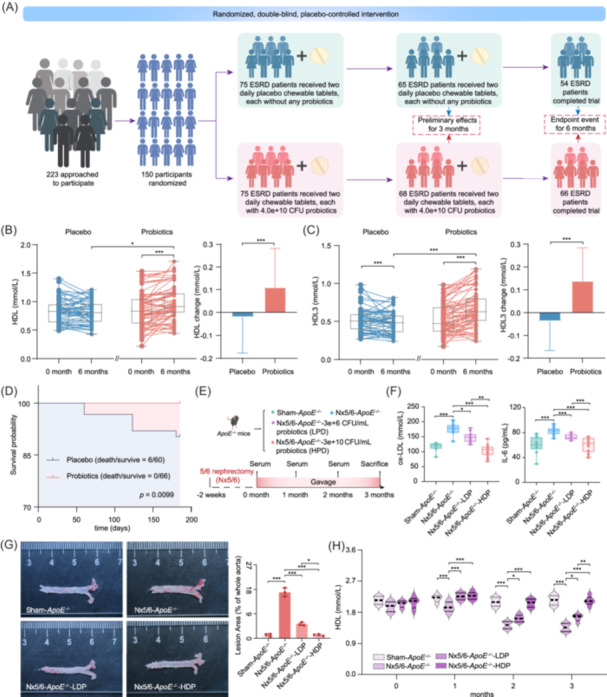
Probiotics improve cardiovascular‐related outcomes in ESRD patients and nephropathic *ApoE^–/^^–^* mice by elevating HDL levels. (A) Flowchart detailing participant recruitment, randomization, follow‐up, and analysis populations in the ESRD study. Changes in (B) HDL and (C) HDL3 after 6 months of probiotic intervention in ESRD. Lines represent raw values before and 6 months after receiving treatment. The distribution of values within each group for each timing is illustrated by a box and whisker. In the box plots, the line in the middle of the box is plotted at the median, the inferior and superior limit of the box correspond to the 25th and the 75th percentiles, respectively. The whiskers correspond to the minimum and maximum values. Wilcoxon matched‐pairs signed‐rank test or paired *t*‐test was performed to verify changes from baseline (intra‐group changes), according to the distribution, when drawn, the capped line above the concerned group shows the corresponding *p*‐value. When the difference is significant, a capped line is marked above the concerned group. Changes between 0 and 6 months across the groups were analysed with Kruskal–Wallis or One‐way ANOVA according to the distribution, respectively. When the difference is significant, a line is marked above the concerned groups with the corresponding *p*‐value. Bars represent mean change from baseline value per group, with their standard deviation. Mann–Whitney *U* test or unpaired *t*‐tests were performed to compare differential values of both probiotics versus the placebo group (intergroup changes), according to the distribution. The respective *p*‐value is indicated in the table, and when the test is significant, bars are marked by * symbol. A total of 120 patients completed the 6‐month intervention period (placebo: probiotics = 54:66). Asterisks indicated significant differences between the two groups: *: *p* < 0.05; ***: *p* < 0.001. (D) Kaplan–Meier curves depicting cardiac death outcomes between placebo and probiotic groups during the 6‐month intervention. (E) Animal experimental design. (F) Concentrations of ox‐LDL and IL‐6 in mice after 3 months of probiotic gavage (Sham, *n* = 10; Nx5/6, *n* = 15; Nx5/6‐LDP, *n* = 13; Nx5/6‐HDP, *n* = 18). (G) Changes in arterial AS plaque distribution in mice after 3 months of probiotic gavage (*n* = 3). Left: Oil red O staining in the aortic arch with four groups. Right: Quantification of staining levels in left. (H) Concentrations of HDL in mice during 3 months of gavage probiotics (*n* = 6). Significant differences between groups are denoted by asterisks: *: *p* < 0.05; **: *p* < 0.01; ***: *p* < 0.001. The *p* values in animal experiments, for comparisons of more than two groups, the *LSD* test or *Tamhane* test in ANOVA was used. Mann–Whitney *U* test was used for non‐normally distributed data. ESRD, end‐stage renal disease; HDL, high‐density lipoprotein.

To assess the clinical impact of probiotics in ESRD, we conducted a 6‐month randomized, double‐blind trial involving 150 patients (75 each in the probiotic group, 75 in the group) (Figure 1A). Baseline characteristics were well‐balanced between the groups (Table S1). Notably, the placebo group exhibited a higher dropout rate (28% vs. 12%) primarily due to adverse events or mortality (Tables S2 and S3). After 3 months, the probiotic group showed significant reductions in oxidative stress and inflammatory markers (CRP, C‐reactive protein; IL‐6, interleukin 6, etc.) and cardiovascular risk indices (atherosclerotic index, cTnT, etc.), which coincided with a significant recovery of HDL to healthy levels and a significant increase in HDL3 (*p* < 0.001) (Tables S4 and S5). By 6 months, CRP and the atherosclerotic index remained lower in the probiotic group, with no cardiovascular deaths reported compared to six deaths in the placebo group (*p* < 0.05). This sustained benefit was accompanied by continued significant increases in HDL and HDL3 (*p* < 0.001) (Figure 1B–D and Tables S4, S6, and S7). Spearman analysis confirmed a significant inverse correlation between HDL and both CRP and the atherosclerotic index (CRP: *r* = −0.324, *p* < 0.001; atherosclerotic index: *r* = −0.762, *p* < 0.0001) (Figure S1), suggesting that HDL‐driven improvements contributed to the enhanced cardiovascular prognosis. These findings are consistent with a meta‐analysis among 30‐year cohort studies, which affirmed the protective role of HDL in ESRD prognosis, demonstrating a pooled hazard ratio of 0.84 (95% CI: 0.77–0.91, *p* < 0.001) for cardiovascular outcomes associated with HDL (Figure S2A). Importantly, other lipid parameters (LDL, low‐density lipoprotein; TC, total cholesterol; and TG, triglycerides) showed no significant associations (Figures S2B and S3), highlighting HDL as a specific therapeutic target in ESRD.

Given the challenges of assessing cardiovascular‐related imaging changes in ESRD within a short timeframe [9], we conducted animal experiments to further investigate the cardiovascular protective effects of probiotics in nephropathy. Utilizing a 5/6 nephrectomy model combined with *ApoE* knockout mice (*ApoE* ^*–/–*^) to mimic lipid alterations observed in nephrotic states (Figure 1E and Figure S4), we observed that probiotic administration led to reduced levels of pro‐atherogenic ox‐LDL and IL‐6, and suppressed early arterial plaque formation (*p* < 0.001) (Figure 1F–G and Figure S5A). High‐dose probiotics also restored cardiac function, as indicated by a significantly higher ejection fraction (62.3 ± 4.1% vs. 45.8 ± 3.9% in the placebo group; *p* < 0.05) (Table S8), suggesting a potential for alleviating dilated cardiomyopathy, a common complication in ESRD patients [10]. Notably, only HDL levels normalized within 1 month in probiotic‐treated mice, reaching levels comparable to non‐nephropathic controls (Figure 1H and Figure S5B–D). These animal study results highlight the rapid and specific HDL‐enhancing effects of probiotics in the context of renal injury, independent of any significant modulation of LDL or TC.

### Intestinal HDL3 synthesis as a novel pathway for probiotic‐mediated cardioprotection in renal injury

**FIGURE 2:**
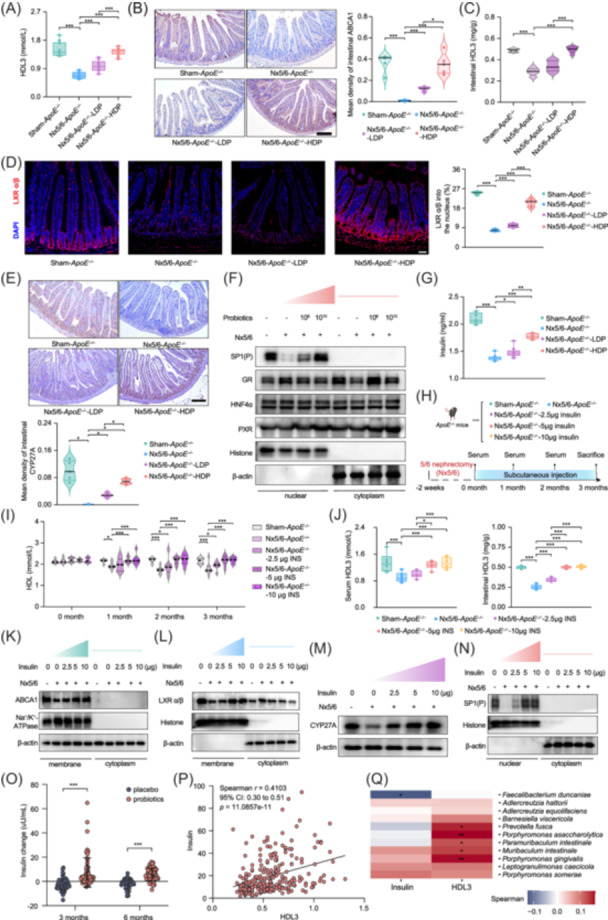
Probiotics modulate the SP1(P)‐CYP27A‐LXR α/β‐ABCA1 pathway in the small intestine by regulating insulin concentrations, thus enhancing HDL3 production. (A) Serum HDL3 concentration in mice after 3 months of probiotic gavage (*n* = 9). (B) Immunohistochemistry of small intestinal ABCA1 expression (scale bar: 200 µm) and its quantification (*n* = 5). (C) Small intestine HDL3 content (*n* = 9). (D) Immunofluorescent staining of LXRα/β expression in four groups (scale bar: 50 µm), with quantification of nuclear localization (*n* = 5). (E) Immunohistochemical staining of CYP27A expression in four groups (scale bar: 200 µm), with quantification of staining levels (*n* = 5). (F) Nuclear CYP27A transcription factor levels were analyzed by immunoblotting, with histone as the nuclear protein loading control and β‐actin as the cytoplasmic control. (G) Serum insulin levels after 3 months of probiotic gavage (*n* = 10). (H) Animal experimental design. (I) Serum levels of HDL after 3 months of insulin injection (*n* = 6). (J) HDL subtype concentrations, including serum HDL3 and small intestinal HDL3 content in mice 3 months after insulin supplementation (*n* = 6). (A–J) Significant differences between groups are denoted by asterisks (*: *p* < 0.05; **: *p* < 0.01; ***: *p* < 0.001). The *p*‐values in animal experiments, for comparisons of more than two groups, the *LSD* test or *Tamhane* test in ANOVA was used. Mann–Whitney *U* test was used for non‐normally distributed data. (K) Expression of small intestinal ABCA1 protein 3 months after insulin injection. Immunoblotting of ABCA1 with Na^+^/K^+^‐ATPase as membrane loading control and β‐actin as cytoplasmic control. (L) Activation of the ABCA1 transcription factor LXRα/β in the small intestine after insulin injection. Immunoblotting of nuclear LXRα/β with histone as nuclear loading control and β‐actin as cytoplasmic control. (M) CYP27A protein expression in the small intestine after insulin injection. Immunoblotting of CYP27A. (N) Activation of CYP27A transcription factor SP1(P) in the small intestine. Immunoblotting of nuclear SP1(P) with histone as nuclear loading control and β‐actin as cytoplasmic control. (O) Serum insulin levels in ESRD patients after 3 and 6 months of probiotics versus placebo. Sample sizes: probiotics group (3 months, *n* = 68; 6 months, *n* = 66), placebo group (3 months, *n* = 65; 6 months, *n* = 54). Significant differences: ***: *p* < 0.001 (Mann–Whitney *U* test). (P) Spearman correlation between insulin and HDL3 levels in ESRD patients (*n* = 253) after probiotics. Correlation coefficient *r* = 0.4103, 95% CI was 0.2989 to 0.5106, *p* < 0.001. (Q) Correlation between the abundance of key differential species in the Sham‐*ApoE^–/–^* and Nx5/6‐*ApoE^–/–^* groups with insulin and HDL3 levels in ESRD patients after 3 months of probiotic treatment. Heatmap shows Spearman correlation coefficients with significance denoted as *: *p* < 0.05; **: *p* < 0.01 (*n* = 133). *ApoE^–/–^*, apolipoprotein E‐deficient mice; CYP27A, cytochrome P450 family 27 subfamily A member 1; GR, glucocorticoid receptor; HDL, high‐density lipoprotein; HDP, high dose probiotics; HNF4α, hepatocyte nuclear factor 4α; INS, insulin; LDP, low dose probiotics; LXR α/β, liver X receptor α/β; Nx5/6, 5/6 nephrectomy; PXR, pregnane X receptor; SP1(P), phosphorylated specificity protein 1.

To elucidate the mechanism underlying probiotic‐induced HDL elevation in severe renal injury, we utilized murine models with 5/6 nephrectomy and *ApoE* ^*–/–*^ deficiency. We observed that probiotics increased the expression of ATP binding cassette subfamily A member 1 (ABCA1), a key transporter for HDL biogenesis, in intestinal epithelial cells, leading to elevated HDL3 levels, but not high‐density lipoprotein 2 (HDL2) (Figure 2A–C and Figure S6A–C). Furthermore, probiotics enhanced small intestine‐derived HDL3 production through increased local HDL synthesis and metabolism, independent of other intestinal cholesterol or liver HDL pathways (Figure S6D–M). Mechanistic investigations revealed that renal injury reduced the nuclear localization of liver X receptors (LXR) α/β in intestinal cells (Figure 2D and Figure S7A,B), which are crucial intestinal regulators of ABCA1 [11]. Probiotics restored LXRα/β nuclear translocation by increasing the levels of intestinal 27‐hydroxycholesterol (Figure S7C), an endogenous LXR ligand produced by cytochrome P450 family 27 subfamily A member (CYP27A) [12]. Analysis of protein and transcript levels showed a downregulation of CYP27A in renal injury, which probiotics reversed in a dose‐dependent manner (Figure 2E and Figure S7D). To understand the factors contributing to decreased CYP27A expression in the small intestine during renal injury, we further screened potential transcriptional regulators. This identified phosphorylated specificity protein 1 (SP1(P)) as a critical regulator of CYP27A, whose nuclear translocation was impaired due to insulin deficiency in nephropathy [13] (Figure 2F,G and Figure S7E). Probiotics restored insulin levels, which are essential for SP1(P) nuclear translocation [14], thereby enabling SP1(P)‐mediated CYP27A transcription. This cascade ultimately enhanced the production of 27‐hydroxycholesterol, which activated LXRα/β to upregulate ABCA1 and promote HDL synthesis (Figure S7F).

The resulting increase in ABCA1 expression via the Insulin‐SP1(P)‐CYP27A‐LXRα/β pathway specifically enhanced HDL3, which is recognized to be more effective than HDL2 in providing primary protection against CVD due to its superior cholesterol removal capacity and anti‐inflammatory and antioxidant properties [15, 16, 17]. This mechanism was found to be specific to renal injury (Figures S8 and S9), with insulin identified as a critical upstream regulator. Supporting this, exogenous insulin administration in *ApoE* ^*–/–*^ mice with renal injury dose‐dependently increased intestinal HDL3 synthesis (*p* < 0.001), mirroring the effects observed with probiotic treatment (Figure 2H–N and Figure S10). Clinically, ESRD patients receiving probiotics exhibited higher serum insulin levels than placebo recipients at both 3 and 6 months (*p* < 0.001), and these insulin levels showed a strong positive correlation with HDL3 concentrations (*r* = 0.4103, 95% CI: 0.2989–0.5106; *p* = 11.0857e‐11) (Figure 2O,P and Figure S11). In summary, our findings indicate that probiotics increase HDL levels in ESRD patients primarily by enhancing intestinal HDL3 production through the insulin‐dependent SP1(P)‐CYP27A‐LXRα/β‐ABCA1 pathway, highlighting the crucial role of intestine health in mitigating cardiovascular risk in these patients.

### Gut microbiota remodeling underlies probiotic efficacy in renal and metabolic dysregulation

A significant difference exists in the gut microbiota composition between patients with ESRD and healthy individuals [18]. Interestingly, insulin secretion is also known to be influenced by gut microbiota [19]. To investigate whether probiotic intervention impacts the gut microbiota in severe kidney injury, we performed high‐throughput shotgun metagenomic sequencing on fecal microbiota samples from both probiotic‐treated mice and patients. Our analysis revealed that high‐dose probiotic treatment shifted the microbial composition in nephropathic mice towards a profile resembling that of non‐nephropathic mice (PERMANOVA; *p* = 0.374) (Figure S12A,B). Specifically, probiotics restored approximately half (30 out of 59 strains) of the microbiota dysbiosis induced by kidney injury, and these restored strains were linked to insulin and HDL3 metabolism (Figure S12C,D).

Shotgun metagenomic sequencing of fecal samples from ESRD patients, however, showed no significant change in overall intestinal flora diversity after 3 months of treatment with either placebo or probiotics (Figure S12E). This lack of significant change in diversity might be attributed to the complex living environment of ESRD patients and the established dysbiotic state of their gut flora, which may require more intensive or prolonged interventions to sufficiently alter [20]. Nevertheless, we confirmed probiotic colonization in treated individuals (Figure S12F). Notably, the bacterial species restored by probiotics in our mouse model also showed a correlation with insulin and HDL3 levels in the human patient cohort (Figure 2Q). These collective results suggest that while the overall diversity in ESRD patients might be resistant to short‐term probiotic intervention, specific probiotic‐induced changes in gut microbiota composition may still influence insulin levels and HDL3 concentrations.

In summary, a comparison of previous studies confirms an inverse relationship between HDL concentrations and the prevalence of CVD. Our work provides the first evidence that probiotics combat cardiovascular risks in ESRD by specifically boosting intestinal HDL3 synthesis—a superior atheroprotective HDL subtype—rather than merely elevating total HDL. The discovery of the insulin‐dependent SP1‐CYP27A‐LXRα/β‐ABCA1 pathway mechanistically links gut microbiota modulation to systemic lipid metabolism, addressing a critical gap in managing ESRD‐related dyslipidemia where statins often fall short. Clinically, this offers a safe, nonpharmacological strategy (probiotics) to potentially reduce CVD mortality in ESRD patients. This dual effect on insulin concentration and HDL3 restoration positions probiotics as a paradigm‐shifting adjunct therapy for renal‐cardio metabolic syndromes.

## CONCLUSION

The observed elevation of HDL3 effectively reduces cardiovascular risk markers and attenuates atherosclerosis progression in both human and murine models of renal injury. Furthermore, probiotics reveal a significant therapeutic potential for improving cardiovascular outcomes in ESRD patients by specifically enhancing HDL3 production via the insulin‐mediated SP1(P)‐CYP27A‐LXRα/β‐ABCA1 pathway.

## METHODS

All the materials and methods are described in the Supporting Information.

## AUTHOR CONTRIBUTIONS

**Xiaoxue Liu**: Conceptualization; investigation; writing—original draft; methodology; validation; visualization; writing—review and editing; software; formal analysis; project administration; data curation; supervision; resources. **Yuan Huang**: Conceptualization; investigation; funding acquisition; methodology; validation; visualization; writing—review and editing; software; formal analysis; project administration; data curation; supervision; writing—original draft. **Yixuan Li**: Conceptualization; investigation; writing—original draft; methodology; validation; visualization; writing—review and editing; software; formal analysis; project administration; data curation; resources; supervision. **Juan Chen**: Conceptualization; investigation; writing—original draft; methodology; validation; visualization; writing—review and editing; software; formal analysis; project administration; data curation; supervision; resources. **Xifan Wang**: Conceptualization; Investigation; writing—original draft; methodology; validation; visualization; writing—review and editing; software; formal analysis; project administration; data curation; supervision; resources. **Xiaobin Wang**: Methodology; data curation; investigation. **Liang Zhao**: Investigation; Methodology; Data curation. **Yongting Luo**: Investigation; Methodology; Validation. **Peng An**: Methodology; data curation; investigation. **Liwei Zhang**: Data curation; formal analysis; software; visualization. **Chengying Zhang**: Data curation; investigation. **Weijing Bian**: Data curation; investigation. **Xingen Lei**: Writing—review and editing. **Xiang Gao**: Writing—review and editing. **Yinghua Liu**: Methodology. **Yanling Hao**: Methodology. **Huiyuan Guo**: Methodology. **Xiaoxu Zhang**: Data curation. **Pengjie Wang**: Data curation. **Ran Wang**: Methodology. **Hao Zhang**: Methodology. **Bing Fang**: Methodology. **Xiaolin Zhang**: Investigation. **Longjiao Wang**: Investigation. **Qinglu Qiu**: Investigation. **Yuchan Zhang**: Investigation. **Jingyi Qi**: Data curation. **Songtao Yang**: Methodology; data curation; supervision; project administration; writing—review and editing; investigation; conceptualization. **Yulong Yin**: Writing—review and editing; data curation; methodology; investigation; supervision; project administration; conceptualization. **Fazheng Ren**: Conceptualization; writing—original draft; methodology; writing—review and editing; project administration; data curation; supervision; resources; investigation; data curation. **Xiaoyu Wang**: Conceptualization; funding acquisition; writing—original draft; writing—review and editing; methodology; project administration; data curation; supervision; resources.

## CONFLICT OF INTEREST STATEMENT

The authors declare no conflicts of interest.

## ETHICS STATEMENT

This study adhered to the guidelines of the International Conference on Harmonization Good Clinical Practice and the principles of the Declaration of Helsinki. Ethical approval was granted by the Ethics Committees of Aerospace Center Hospital (Aerospace Clinical Medical College of Peking University), China Agricultural University, Beijing Anzhen Hospitals, and the Total Hospital of the Chinese People's Armed Police Force. All participants provided written informed consent before participation, and compensation was provided as outlined in the informed consent form. The trial was registered at ClinicalTrials.gov to ensure transparency and public access to the study's design and outcomes (clinicaltrials.gov, NCT03010735).

## Supporting information


**Figure S1:** Spearman correlation analysis between prognostic indicators and lipid measures (HDL, LDL, TC, TG) in ESRD patients (*n* = 133) after 3 months of probiotic treatment.
**Figure S2:** Population‐based studies of blood lipids: A meta‐analysis of prospective cohort study of HDL (A) and LDL (B) concentrations and diverse clinical endpoint events in ESRD patients.
**Figure S3:** Population‐based studies of blood lipids: A meta‐analysis of prospective cohort study of TC (A) and TG (B) concentrations and diverse clinical endpoint events in ESRD patients.
**Figure S4:** TC, TG, HDL, and LDL concentrations in *ApoE* ^*−/−*^ with severe renal injury and C57BL/6J mice during 3 months after 5/6 nephrectomy.
**Figure S5:** Effects of probiotics supplementation on lipids and CVD in nephropathic *ApoE* ^*−/−*^ mice.
**Figure S6:** Effect of probiotics on cholesterol metabolism in *ApoE* ^*−/−*^ mice with severe renal injury.
**Figure S7:** Probiotics enhance small intestinal ABCA1 expression via the insulin‐mediated SP1(P)‐CYP27A‐LXR α/β‐ABCA1 pathway in nephropathic *ApoE* ^*−/−*^ mice.
**Figure S8:** Blood lipids concentrations in *ApoE* ^*−/−*^ mice during 3 months of probiotic gavage (*n* = 6).
**Figure S9:** Effect of probiotic supplementation on HDL content and synthesis in C57BL/6 J mice.
**Figure S10:** Relation between insulin and HDL content in nephropathic *ApoE* ^*−/−*^ mice.
**Figure S11:** Effect of probiotics on insulin and HbA1c in ESRD patients.
**Figure S12:** Comparative analysis of the gut microbial composition in fecal samples from nephropathic *ApoE* ^*−/−*^ mice or patients with ESRD treated with probiotics.

**Table S1:** Summary on statistics of the host properties and clinical indexes from placebo and probiotics cohorts.
**Table S2:** Withdrawal and adverse event records.
**Table S3:** Withdrawal and adverse event analysis (Chi‐square test).
**Table S4:** Changes of clinical indicators after probiotics intervention.
**Table S5:** Changes in atherosclerosis index over 3 months (Chi‐square test).
**Table S6:** Changes in atherosclerosis index over 6 months (Chi‐square test).
**Table S7:** Comparison of deaths in the two groups over 6 months (Chi‐square test).
**Table S8:** Echocardiographic results at the endpoint of intervention in mice.

## Data Availability

The paper, along with its extended data and supplementary materials, contains all the data necessary for evaluating the conclusions presented. Additionally, datasets that support the findings of this study can be obtained from the corresponding author upon a reasonable request. In cases where data sharing is permissible, the information will be disseminated under a material transfer agreement. The source data from the ESRD‐related clinical studies are not publicly available due to legal and consent restrictions. Requests to access the data sets should be directed to the study's principal investigators, Xiaoyu Wang (xy.wang@cau.edu.cn) and Fazheng Ren (renfazheng@cau.edu.cn). If access is granted, users must adhere to a data access agreement, which includes responsibilities regarding third‐party data sharing, maintaining participant privacy, and acknowledging the data source. The fecal macrogenome data can be found at NCBI under BioProject accession number: PRJNA1272654 (https://www.ncbi.nlm.nih.gov/sra/PRJNA1272654). The data and scripts used are saved in GitHub https://github.com/Liuxiaoxue0828/ESRD-Pro./tree/main. Supplementary materials (methods, figures, tables, graphical abstract, slides, videos, Chinese translated version, and update materials) may be found in the online DOI or iMeta Science http://www.imeta.science/.
