# Supplementary material for: Probiotics restore enteric HDL3 secretion and improve prognosis in patients with end‐stage renal disease
Source: Imeta. 2025 Jul 1;4(4):e70062. doi: 10.1002/imt2.70062 (PMC12371253; doi:10.1002/imt2.70062)
Supplement: Supplementary file 1 — Figure S1: Spearman correlation analysis between prognostic indicators and lipid measures (HDL, LDL, TC, TG) in ESRD patients (n = 133) after 3 months of probiotic treatment. Figure S2: Population‐based studies of blood lipids: A meta‐analysis of prospective cohort study of HDL (A) and LDL (B) concentrations and diverse clinical endpoint events in ESRD patients. Figure S3: Population‐based studies of blood lipids: A meta‐analysis of prospective cohort study of TC (A) and TG (B) concentrations and diverse clinical endpoint events in ESRD patients. Figure S4: TC, TG, HDL, and LDL concentrations in ApoE −/− with severe renal injury and C57BL/6J mice during 3 months after 5/6 nephrectomy. Figure S5: Effects of probiotics supplementation on lipids and CVD in nephropathic ApoE −/− mice. Figure S6: Effect of probiotics on cholesterol metabolism in ApoE −/− mice with severe renal injury. Figure S7: Probiotics enhance small intestinal ABCA1 expression via the insulin‐mediated SP1(P)‐CYP27A‐LXR α/β‐ABCA1 pathway in nephropathic ApoE −/− mice. Figure S8: Blood lipids concentrations in ApoE −/− mice during 3 months of probiotic gavage (n = 6). Figure S9: Effect of probiotic supplementation on HDL content and synthesis in C57BL/6 J mice. Figure S10: Relation between insulin and HDL content in nephropathic ApoE −/− mice. Figure S11: Effect of probiotics on insulin and HbA1c in ESRD patients. Figure S12: Comparative analysis of the gut microbial composition in fecal samples from nephropathic ApoE −/− mice or patients with ESRD treated with probiotics. [file IMT2-4-e70062-s001.docx]

**Supporting information to**

**Probiotics Restore Enteric HDL3 Secretion and Improve Prognosis in Patients with End-Stage Renal Disease**

**Running title: Probiotics Restore Enteric HDL3 Secretion and Improve ESRD Prognosis**

Xiaoxue Liu^1^*, Yuan Huang^2^*, Yixuan Li^1^*, Juan Chen^1^*, Xifan Wang^3^*, Xiaobin Wang^4^, Liang Zhao^5^, Yongting Luo^1^, Peng An^1^, Liwei Zhang^1^, Chengying Zhang^6^, Weijing Bian^7^, Xingen Lei^8^, Xiang Gao^9^, Yinghua Liu^10^, Yanling Hao^1^, Huiyuan Guo^1^, Xiaoxu Zhang^1^, Pengjie Wang^1^, Ran Wang^1^, Hao Zhang^5^, Bing Fang^1^, Xiaolin Zhang^5^, Longjiao Wang^1,11^, Qinglu Qiu^5^, Yuchan Zhang^5^, Jingyi Qi^1^, Songtao Yang^12#^, Yulong Yin^13,14,15#^, Fazheng Ren^1,11#^, Xiaoyu Wang^1,5#^

^1^Key Laboratory of Precision Nutrition and Food Quality, Department of Nutrition and Health, China Agricultural University, Beijing, China.

^2^Cardiac Surgery Centre, Fuwai Hospital, National Center for Cardiovascular Diseases, Chinese Academy of Medical Sciences, Peking Union Medical College, Beijing, China.

^3^Department of Obstetrics and Gynecology, Columbia University, New York, USA.

^4^Division of General Pediatrics & Adolescent Medicine, Department of Pediatrics, Johns Hopkins University School of Medicine, Baltimore, Maryland, USA.

^5^College of Food Science & Nutritional Engineering, China Agricultural University, Beijing, China.

^6^Department of General Practice, The Third Medical Center of Chinese PLA General Hospital (Total Hospital of the Chinese People’s Armed Police Force), Beijing, China.

^7^Department of Nephrology, Beijing Anzhen Hospital, Capital Medical University, Beijing, China.

^8^Department of Animal Science, Cornell University, Ithaca, New York, USA.

^9^Department of Nutrition and Food Hygiene, School of Public Health, Institute of Nutrition, Fudan University, Shanghai, China.

^10^Department of Nutrition, The First Medical Center of Chinese PLA General Hospital, Beijing, China.

^11^Food Laboratory of Zhongyuan, Luohe, China.

^12^China Aerospace Science & Industry Corporation 731 Hospital, Beijing, China.

^13^Yuelushan Laboratory, Changsha, China

^14^Key Laboratory of Livestock and Poultry Resources (Pig) Evaluation and Utilization, Ministry of Agriculture and Rural Affairs, College of Animal Science and Technology, Hunan Agricultural University, Changsha, China

^15^Institute of Subtropical Agriculture, Chinese Academy of Sciences, Changsha, China

* These authors contributed equally to this work.

Correspondence

Songtao Yang, China Aerospace Science & Industry Corporation 731 Hospital, Beijing, 100074, China.

Yulong Yin, Yuelushan Laboratory, Changsha, 410125, China; Key Laboratory of Livestock and Poultry Resources (Pig) Evaluation and Utilization, Ministry of Agriculture and Rural Affairs, College of Animal Science and Technology, Hunan Agricultural University, Changsha, 410128, China; Institute of Subtropical Agriculture, Chinese Academy of Sciences, Changsha, 410125, China.

Fazheng Ren, Key Laboratory of Precision Nutrition and Food Quality, Department of Nutrition and Health, China Agricultural University, Beijing, 100091, China; Food Laboratory of Zhongyuan, Luohe, 462000, China.

Xiaoyu Wang, Key Laboratory of Precision Nutrition and Food Quality, Department of Nutrition and Health, China Agricultural University, Beijing, 100091, China; College of Food Science & Nutritional Engineering, China Agricultural University, Beijing, 100193, China.

**Supplementary methods**

1. **Sample Size Calculation Basis**

1) Expected Effect Size

● Cardiovascular Events: Literature indicates that the incidence of cardiovascular disease (CVD) events in ESRD patients is 80%[1]. Thus, the placebo group’s CVD event rate is 80%. We hypothesize that the probiotics group will exhibit a 20% reduction in CVD event rate compared to the placebo group.

2) α Error Rate (Significance Level)

● α = 0.05: This is the conventional significance level, indicating a 5% probability of falsely rejecting the null hypothesis when it is true.

3) β Error Rate (Test Power)

● β = 0.20: This represents a 20% probability of falsely accepting the null hypothesis when it is false, corresponding to a statistical power of 1−β=80%1−*β*=80%.

4) Sample Size Calculation for Cardiovascular Events

● Formula: The sample size for comparing two independent proportions is calculated using:


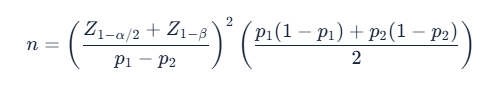


*p*1​ and *p*2​ are the CVD event rates in the two groups.

● Parameter Estimates:

- *p*1​=0.8 (placebo group)
- *p*2​=0.64 (probiotics group)
- Z1−α/2=1.96 (for α = 0.05)
- Z1−β=0.84 (for β = 0.20)

● Calculation: n=59.78

Each group requires 60 patients, totaling 120 patients.

5) Sample Size Adjustment

● Lost to Follow-Up Rate: Assuming a 20% lost-to-follow-up rate, the sample size is adjusted to account for attrition.

- Cardiovascular Events: The total sample size is adjusted to 120*(1−0.20) = 150, requiring 75 patients per group and 150 patients in total.

6) Conclusion

● Cardiovascular Events: To detect the hypothesized effect of probiotics on reducing CVD events, 75 patients per group (total 150 patients) are required.

1. **Human population recruitment**

This double-blind, placebo-controlled, randomized clinical trial was conducted at three hospitals in Beijing, China, to evaluate the efficacy of a novel intervention in ESRD patients undergoing stable hemodialysis (three times a week). The study followed the Kidney Disease: Improving Global Outcomes Clinical Practice guidelines. Participants were excluded if they had used antibiotics or probiotic products in the past 30 days or had a significant allergy to skim milk powder. A total of 150 hemodialysis patients (aged 21–70 years, 48 women) were recruited. Among the ESRD patients, 59 (39.3%) had glomerulonephritis, 43 (28.7%) had diabetic nephropathy, 18 (12%) had hypertensive nephrosclerosis, 7 (4.7%) had polycystic kidney disease, 10 (6.7%) had interstitial nephritis, and 13 (8.6%) had kidney failure of unknown etiology. By the end of the three-month study period, 133 participants were enrolled, and 122 successfully completed both phases of the trial.

1. **Human randomization and masking**

Randomization (1:1) was employed to allocate ESRD patients into two groups, receiving either probiotics or a placebo. Stratification was implemented based on sex, age, protopathic diseases, complications, smoking, drinking, dietary pattern, and membrane type of dialysis. Both groups exhibited comparable baseline indicators (Table S1). To ensure unbiased assessment, patients, care providers, investigators, and outcome assessors were kept blinded to the group allocation.

1. **Procedures**

The probiotic group received two daily chewable tablets, each containing 4.0e+10 CFU of probiotics (*Bifidobacterium animalis subsp. lactis* A6, *Lactobacillus rhamnosus* GG, *Lactobacillus acidophilus* A5, and *Lactobacillus paracasei* L9, 2:1:1:1). The placebo group received two identical-looking chewable tablets with skim milk excipients. The trial lasted six months, with assessments at the start, mid-point, and end. Blood parameters were evaluated at three months to assess the preliminary effects of the probiotics. The endpoint, cardiovascular mortality, was monitored until death or the end of the study (Figure 1A).

1. **Clinical indicators analyses**

Blood samples were procured from patients immediately before hospital-based hemodialysis sessions. Concentrations of key blood biochemical analytes, including Total Cholesterol (TC), High-Density Lipoprotein (HDL), Low-Density Lipoprotein (LDL), Triglycerides (TG), and other relevant markers, were retrieved from the hospital's laboratory information system. We did not measure HDL3 via ultracentrifugation but sedimentation because of insufficient plasma[2,3]. HDL was separated by precipitating LDL and very-low-density lipoprotein cholesterol using dextran sulfate and magnesium chloride. HDL3 was isolated by precipitating HDL2 from total HDL by increasing the concentrations of these reagents. The amount of HDL2 was calculated by subtracting HDL3 from HDL. Atherosclerosis index (AI) was calculated according to the following formula: AI = (TC − HDL)/HDL. Additional indicators were assayed in the laboratory. For the analysis, blood samples were permitting coagulation for at least 1 hour, followed by centrifugation at 3000g for 15 minutes. The resultant serum was subdivided into aliquots, cryopreserved at -80°C. These samples were thawed to measure Cardiac Troponin T (cTnT) concentrations utilizing a third-generation assay (ElecSys 2010 system, Roche Diagnostics, Switzerland). Besides, insulin concentrations were assessed using an ELISA kit (ALPCO, USA). Quantification of IL-6, VCAM, and ox-LDL was conducted through enzyme-linked immunoassay (Multi Sciences, China). And HbA1c was measured by glycosylated hemoglobin content assay kit (Boxbio, China).

1. **Meta-analysis**

This meta-analysis reviewed cohort studies examining blood lipid profiles in ESRD patients with cardiovascular, cerebrovascular, or clinical mortality events. The DerSimonian-Laird method was used for comprehensive analysis, incorporating both controlled terms (MeSH terms from PubMed) and free text search terms. References were sourced from PubMed and Web of Science, covering the period from January 1, 1993, to January 1, 2023. Animal studies, unrepresentative population samples, and studies lacking lipid profiles or clinical endpoint data were excluded. Cohort studies using lipid indicators to assess cardiovascular health events, including myocardial infarction, coronary artery disease, cerebral infarction, and mortality, were included. Studies were weighted based on the precision of effect size estimates, favoring more accurate studies. Despite the heterogeneity of the included studies (Figure S2 and S3), no consistent patterns were found to justify adjustments in their combined estimates or to significantly correlate with the varied study characteristics. A total of nine articles were used for evaluating HDL, seven for total cholesterol (TC) and triglycerides (TG), and eight for LDL[4−14].

1. **Animal experiments**

All animal procedures were ethically approved by the Ethics Committee of China Agricultural University (approval numbers: AW62051202-4-1 and AW51203202-5-1). Six-week-old male C57BL/6J and *ApoE*^–/–^ mice (specific pathogen-free grade, Beijing Hua Fukang Biotechnology Co., Ltd., Beijing, China) were housed in filter-top cages with a strict 12-hour light/dark cycle and had ad libitum access to standard chow and water. After a one-week acclimation period, a 5/6 nephrectomy was performed to create a model of CKD[15]. Mice were randomly assigned to probiotic treatment or validation groups two weeks after surgery.

*Probiotic Intervention Study 1*:

In this study, *ApoE*^–/–^ mice were used to address the challenge of inducing dyslipidemia and cardiovascular conditions. The mice were divided into four groups: control (Sham-*ApoE*^–/–^), nephropathic (Nx5/6-*ApoE*^–/–^), nephropathic + lowdose probiotics (Nx5/6-*ApoE*^–/–^-LDP), and nephropathic + high-dose probiotics (Nx5/6-*ApoE*^–/–^-HDP) mice. Mice received daily gavage of 200 µL of mixed probiotics (high dose: 3.0e+10 CFU/mL, low dose: 3.0e+6 CFU/mL) in normal saline for three months (Figure 1E).

*Probiotic Intervention Study 2*:

In this study, *ApoE*^–/–^ mice were divided into two groups: control (*ApoE*^–/–^) and probiotics (Pro.-*ApoE*^–/–^). The probiotics group received daily oral administration of 200 µL of mixed probiotics (3.0e+10 CFU/mL) in normal saline for three months (Figure 8A).

*Probiotic Intervention Study 3*:

C57BL/6J mice were divided into three groups: sham-operated (Sham-WT), nephropathic (Nx5/6-WT), and nephropathic + probiotics (Nx5/6-Pro.-WT). The probiotics group received daily oral administration of 200 µL of mixed probiotics (3.0e+10 CFU/mL) for three months (Figure S9A).

*Validation Study*:

In the validation study, *ApoE*^–/–^ mice were divided into five groups: control (Sham-*ApoE*^–/–^), nephropathic mice (Nx5/6-*ApoE*^–/–^), nephropathic + insulin 2.5 μg (Nx5/6-*ApoE*^–/–^-2.5 μg INS), nephropathic + insulin 5 μg (Nx5/6-*ApoE*^–/–^-5 μg INS), and nephropathic + insulin 10 μg (Nx5/6-*ApoE*^–/–^-10 μg INS). Insulin was administered daily via subcutaneous injection over a three-month period (Figure 2H).

1. **Animal sample collections**

Pre- and post-probiotics or insulin interventions, murine blood samples were obtained at 0, 1, 2, and the final 3 months for serum isolation, preserved at -80°C for subsequent analyses. Upon study completion, fecal and tissue specimens were collected. Animals were euthanized through cervical dislocation under ether anesthesia. Half of the tissue samples were promptly frozen in liquid nitrogen and stored at -80°C for further analyses, while the remaining half underwent fixation in 10% formalin for histological and immunohistochemical assessments.

1. **Metabolic indicators in mice**

The laboratory quantified TG, TC, LDL, and HDL concentrations in mice serum using a BS-420 automated biochemical analyzer. And mouse insulin concentrations were assessed using an ELISA kit (ALPCO, USA). Quantification of ox-LDL and IL-6 were conducted through enzyme-linked immunoassay (Multi Sciences, China). Besides, we did not measure HDL3 via ultracentrifugation but sedimentation because of insufficient plasma. And intestinal HDL subtypes distribution in mice were determined through ELISA kit (Raysun Biotechnology, China). And intestinal oxysterols were quantitated by referencing to the spiked internal standards as previously described[16]. Fecal cholesterol distribution in mice was analyzed using commercial kits from Nanjing Jiancheng Bioengineering Institute, China. Total cholesterol (Nanjing Jiancheng Bioengineering Institute, China) and free cholesterol (Solarbio, China) distribution in mouse tissues were examined using commercial kits.

1. **Tissue protein distribution assessment**

*Histological and* *immunohistochemical analyses*: For tissue protein distribution assessment, 10%-formaldehyde-fixed tissues underwent dehydration using a graded ethanol series at room temperature, followed by paraffin embedding and sectioning into 5 μm slices. Immunohistochemical staining involved microwave heating of sections in 0.01 M citrate buffer (pH 6) at 95 °C for 10 minutes to repair antigens. Subsequently, sections underwent immunostaining using the ABC peroxidase method (Vector Laboratories, USA) with diaminobenzidine as the enzyme substrate and hematoxylin for counterstaining. For immunofluorescence staining, paraffin sections were microwave-treated, incubated with primary antibodies, followed by incubation with secondary antibodies (Invitrogen, USA), and counterstained with DAPI within a mounting medium. The antibody used was ABCA1 (Abcam, USA), Apo A (Abcam, USA), SRBⅠ (Abcam, USA), CD36 (Proteintech, USA), LXR α/β (Santa cruz, USA), CYP27A (Proteintech, USA), and LIPA (Proteintech, USA),

*Western blot*: To evaluate tissue protein levels comprehensively, tissue proteins were extracted using nuclear or membrane protein extraction kits. Protein concentrations were determined using standard BCA methods. Proteins were resolved on SDS-PAGE gels for electrophoresis and transferred to PVDF membranes. Following skimmed milk sealing, membranes were incubated with primary antibodies overnight at 4°C. Subsequently, membranes were washed with PBST and incubated with a horseradish peroxidase-conjugated secondary antibody. Subsequently, the membrane was treated with enhanced chemiluminescence compound treatment solution and photographed and counted with a gel imager. The antibody used was ABCA1 (Abcam, USA), LXR α/β (Santa cruz, USA), CYP27A (Proteintech, USA), GR/NR3C1 (Novus USA), PXR (Abcam, USA), SP1(P) (Santa cruz, USA), HNF4α (Abcam, USA), ATP1A1 (Proteintech, USA), Histone3 (Proteintech, USA), and β-actin (Proteintech, USA).

1. **Real-time quantitative PCR analysis**

Quantitative PCR analyses were performed using the SYBR Green qPCR Master mix (Thermo Scientific) and the LightCycler 96 Real-Time PCR System. Primer sequences were as follows: ABCA1 forward: 5′- GCTTGTTGGCCTCAGTTAAGG -3′, ABCA1 reverse: 5′- GTAGCTCAGGCGTACAGAGAT -3′. CYP27A forward: 5′- CCAGGCACAGGAGAGTACG -3′, CYP27A reverse: 5′- GGGCAAGTGCAGCACATAG -3′.

1. **Evaluation of cardiovascular disease in mice**

*Extent of Atherosclerotic Lesions*: The mouse aorta was excised, and consecutive 10μm transverse sections of the aortic arch were acquired and assessed for lipid distribution in the vessel's transverse area via oil red O staining. Simultaneously, the entire aorta underwent global oil red O staining to observe the comprehensive distribution of lipid plaques throughout the arteries.

*Transthoracic echocardiography*: We conducted serial in vivo echocardiography in lightly anesthetized mice (heart rate ≥ 500 bpm) while maintaining a body temperature of 37°C, employing Vevo 3100 (FUJIFILM VisualSonics, Canada). Following the discontinuation of anesthesia, we obtained two-dimensional and M-mode images (parasternal long axis and short axis) of the left ventricle. Cardiac function indices were assessed using M-mode tracings derived from the average of three consecutive heartbeats. All measurements were executed by a seasoned operator who was blinded to the study, ensuring a meticulous and unbiased evaluation of cardiac parameters.

1. **Fecal sample collection and metagenome detection**

Fecal samples were collected in sterile retention bottles and immediately placed on ice. The samples were transported to the laboratory within one hour and frozen at −80°C for later use. Four samples per group were collected from the mice. Among the 150 human participants who completed the trial, 88.7% provided complete fecal samples after three months of treatment. Fecal metagenomes were analyzed using whole-metagenome shotgun sequencing on the Illumina NovaSeq PE150 platform (Illumina Inc., San Diego, CA, USA), performed by Majorbio Bio-Pharm Technology Co., Ltd. (Shanghai, China). Metagenomic sequencing followed previously described methods[17]. In short, total genomic DNA was extracted from human fecal samples using the E.Z.N.A.® Soil DNA Kit (Omega Bio-tek, Norcross, GA, USA), according to the manufacturer's instructions. DNA concentration and purity were determined using the TBS-380 micro fluorometer (Turner Bio Systems, USA) and the NanoDrop2000 ultra-micro spectrophotometer (Thermo Fisher Scientific, USA). Raw sequencing reads underwent quality control using fastp (v0.23.0)[18]. Low-quality reads (those with >45 bases having a quality score < 20 or > 5 ‘N’ bases), low-complexity reads, and adapter-containing reads were removed. Remaining reads were trimmed at the ends for low-quality bases (< Q20) or ‘N’ bases. Human genomic reads were excluded by mapping to the reference human genome (GRCh38) using Bowtie2 (v2.4.4)[19]. The gut microbiota composition was then quantified using MetaPhlAn4 (v4.0.2)[20].

1. **Statistical analysis**

Data were presented as mean ± standard deviation. Outlier detection and data accuracy were ensured using standard plotting and screening techniques. The two-tailed Student’s t-test was used to compare two groups with normal distribution. For comparisons among more than two groups, the LSD test or Tamhane’s test in ANOVA was applied. The Mann-Whitney *U* test was used for non-normally distributed data. Statistically significant differences were defined as *p* < 0.05. Discontinuity frequencies were assessed using the chi-square test. Kaplan-Meier curves were used to represent intervention and survival data. Statistical analyses were performed using STATA 18.0 (Stata Corp., USA), SPSS 22.0 (SPSS, Inc., USA), GraphPad Prism 8.0 (GraphPad, Inc., USA), and R 4.2.2 (survminer 0.4.8, survival 3.2.12).

1. **Graphical abstract**

The structured graphical abstract was created with MedPeer ([www.medpeer.cn](http://www.medpeer.cn)). It is licensed for use and publication by the relevant organizations.


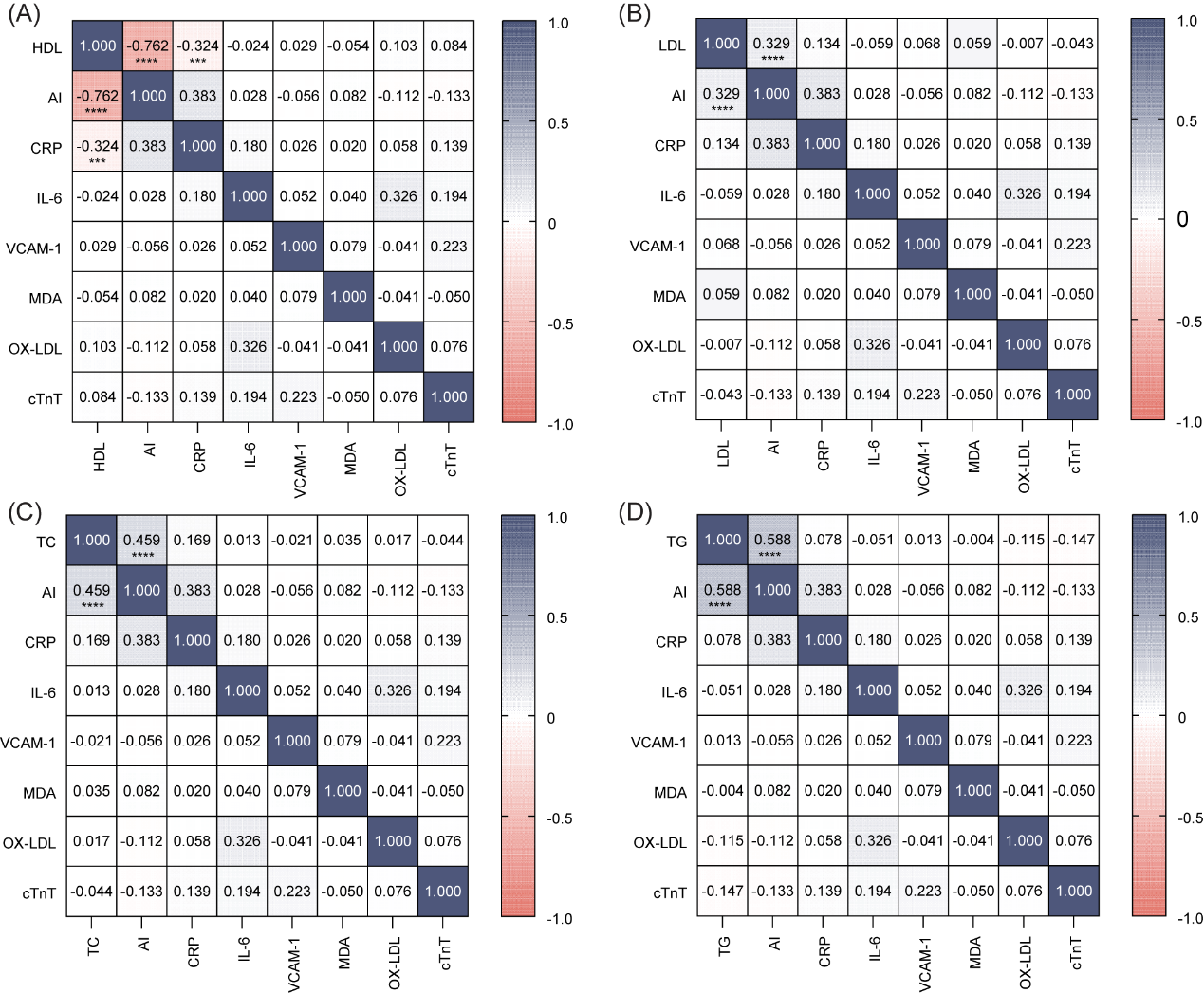


**Figure S1. Spearman correlation analysis between prognostic indicators and lipid measures (HDL, LDL, TC, TG) in ESRD patients (*n =* 133) after 3 months of probiotic treatment.** Panels show correlations for (A) HDL, (B) LDL, (C) TC, and (D) TG. Asterisks indicate significant differences: ***: *p* < 0.001, ****: *p* < 0.0001. HDL, high-density lipoprotein; LDL, low-density lipoprotein; TC, total cholesterol; TG, triglycerides; AI, atherosclerosis index; CRP, C-reactive protein; IL-6, interleukin-6; VCAM-1, vascular cell adhesion molecule 1; MDA, malondialdehyde; ox-LDL, oxidized low-density lipoprotein; cTnT, cardiac troponin T.


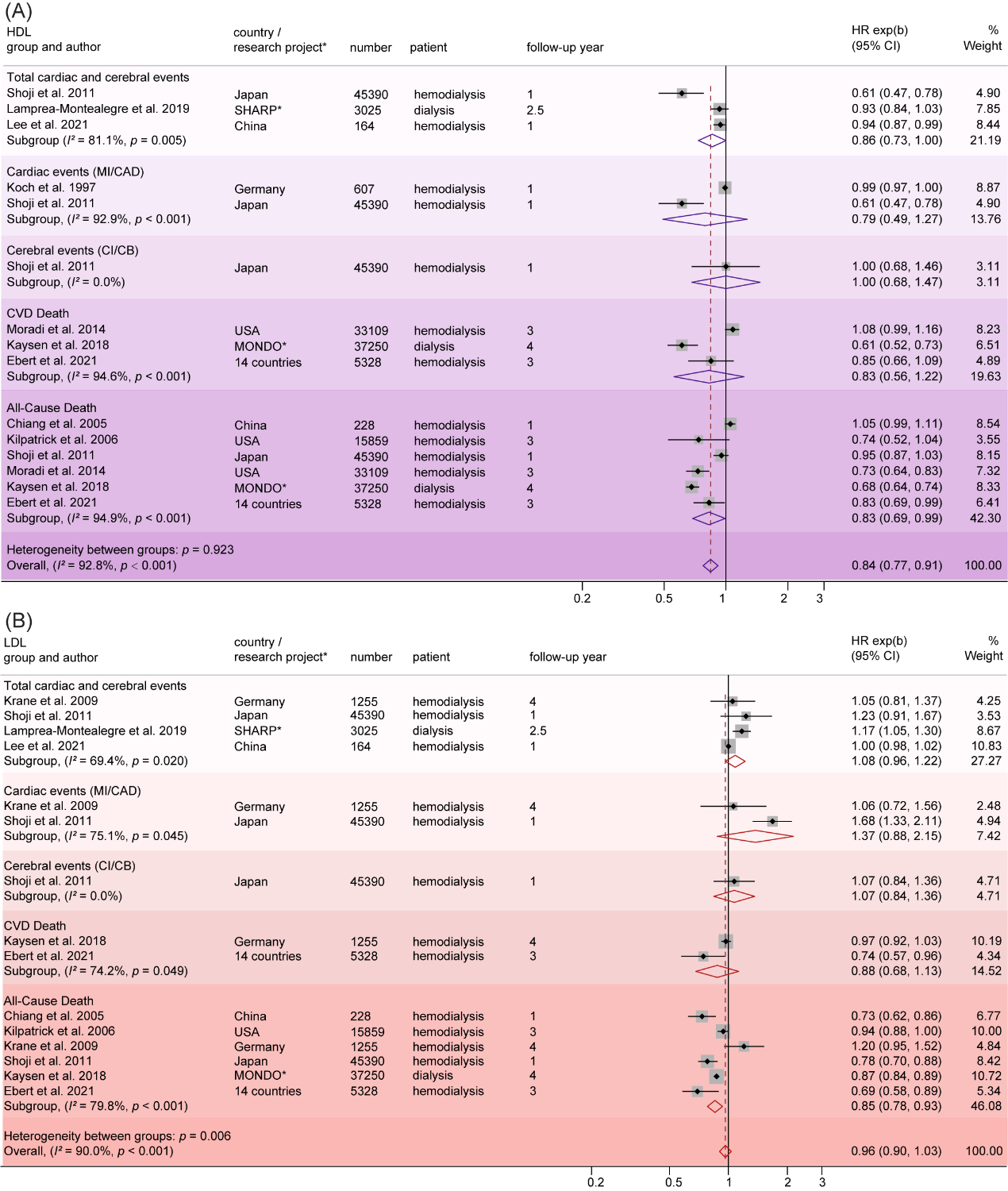


**Figure S2. Population-based studies of blood lipids A meta-analysis of prospective cohort study of HDL (A) and LDL (B) concentrations and diverse clinical endpoint events in ESRD patients.** All documents included were cohort studies. The DerSimonian-Laird method was used to conduct a comprehensive analysis. HR, hazard ratio. The figure featured a central vertical line valued at 1, representing the no-difference line, with outcome effect sizes from the trials displayed horizontally. The overall effect size was shown as a small diamond. Each horizontal line indicated the distribution of effect sizes, and crossing or touching the no-difference line suggested no statistically significant difference between lipid indicators and ESRD patient outcomes. The forest plot's right side displayed each study's weight, based on sample size and effect size estimates—larger sample sizes and more accurate estimates yield higher weights. In the lower left corner, the *I²* and *p* values indicated the heterogeneity among the included studies. ESRD, end-stage renal disease; HDL, high-density lipoprotein; LDL, low-density lipoprotein; MI, myocardial infarction; CAD, coronary artery disease; CI, cerebral infarction; CB, cerebral bleeding; CVD, cardiovascular disease.


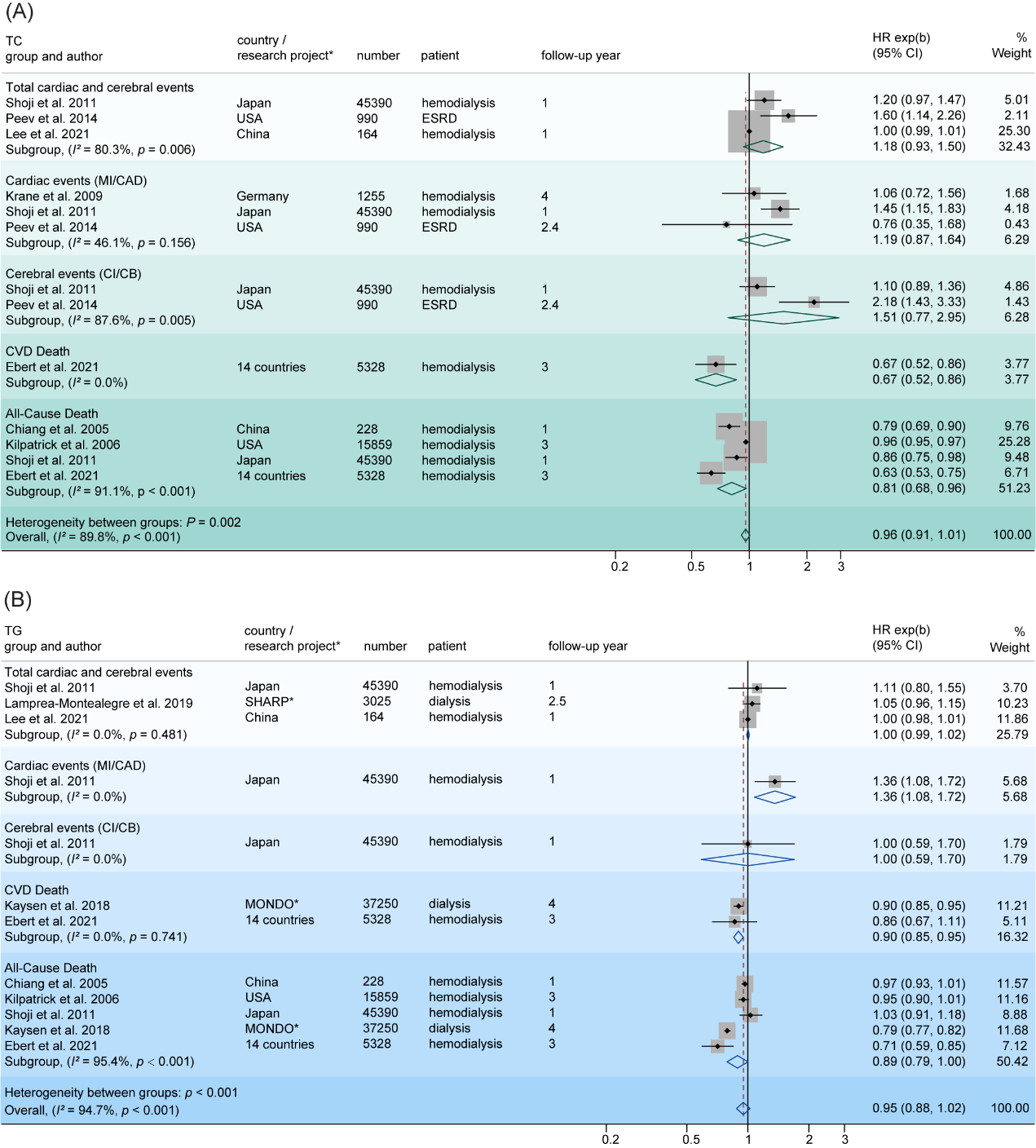


**Figure S3. Population-based studies of blood lipids A meta-analysis of prospective cohort study of TC (A) and TG (B) concentrations and diverse clinical endpoint events in ESRD patients.** All documents included were cohort studies. The DerSimonian-Laird method was used to conduct a comprehensive analysis. HR, hazard ratio. The figure featured a central vertical line valued at 1, representing the no-difference line, with outcome effect sizes from the trials displayed horizontally. The overall effect size was shown as a small diamond. Each horizontal line indicated the distribution of effect sizes, and crossing or touching the no-difference line suggested no statistically significant difference between lipid indicators and ESRD patient outcomes. The forest plot's right side displayed each study's weight, based on sample size and effect size estimates—larger sample sizes and more accurate estimates yield higher weights. In the lower left corner, the *I²* and *p* values indicated the heterogeneity among the included studies. ESRD, end-stage renal disease;TC, total cholesterol; TG, triglycerides; MI, myocardial infarction; CAD, coronary artery disease; CI, cerebral infarction; CB, cerebral bleeding; CVD, cardiovascular disease.


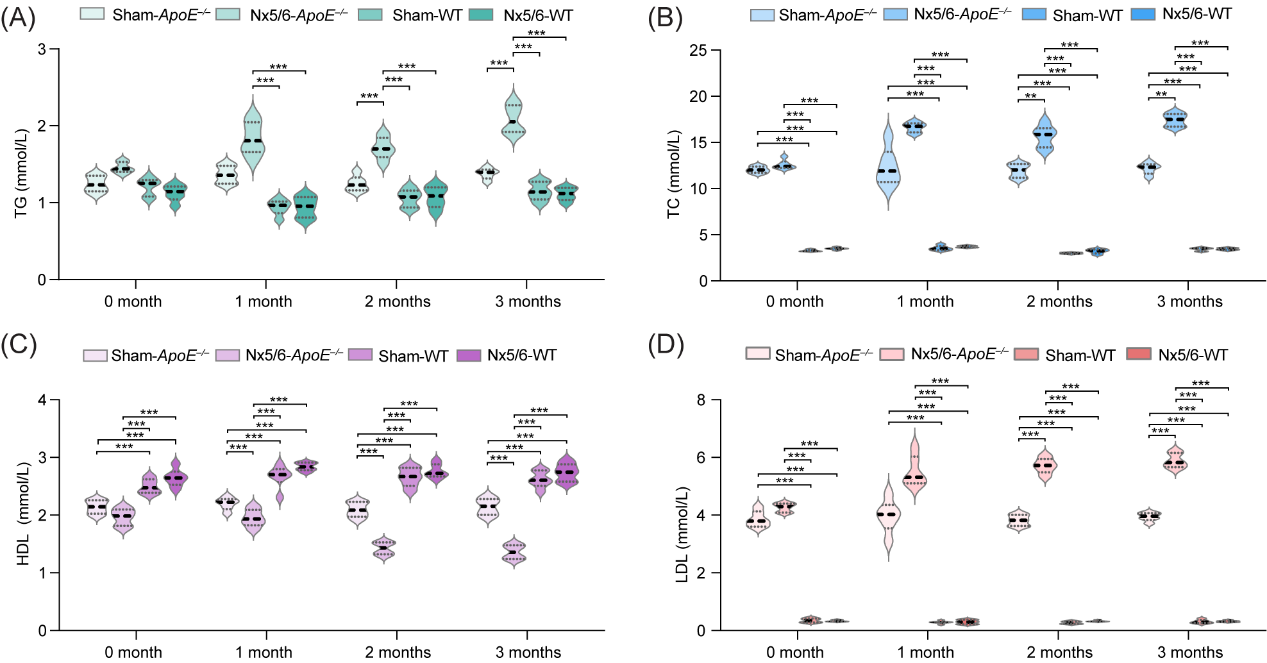


**Figure S4. TC, TG, HDL, and LDL Concentrations in *ApoE^-/-^* with severe renal injury and C57BL/6J mice during 3 months after 5/6 nephrectomy.** *n =* 6, asterisks indicated significant differences between the two groups: ***: *p* < 0.001. The *p*-values in animal experiments, for comparisons of more than two groups, the *LSD* test or *Tamhane* test in ANOVA was used. Mann-Whitney *U* test was used for non-normally distributed data. *ApoE⁻/⁻*, apolipoprotein E-deficient mice; Nx5/6, 5/6 nephrectomy; WT, wild type mice; TG, triglycerides; TC, total cholesterol; HDL, high-density lipoprotein; LDL, low-density lipoprotein.


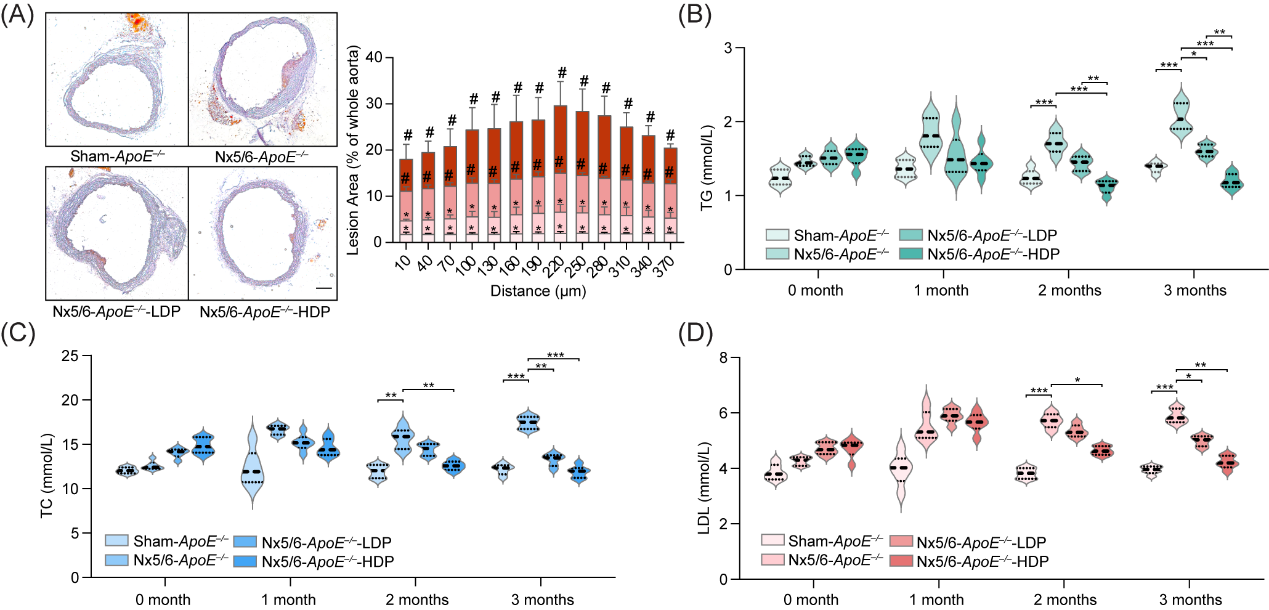


**Figure S5. Effects of probiotics supplementation on lipids and CVD in nephropathic *ApoE^-/-^* mice.** (A) Changes in arterial AS plaque accumulation in mice after 3 months of probiotic gavage (*n =* 3). Left: Oil red O staining in the aorta with four groups. Right: Quantification of staining levels in left. With wells indicating significant differences between and sham-operated group and asterisks indicating significant differences between and Nx5/6 group. Scale bars: 200 µm. (B−D) Concentrations of TG, TC and LDL in mice during 3 months of gavage probiotics (*n =* 6). Significant differences between groups are denoted by asterisks: *: *p* < 0.05; **: *p* < 0.01; ***: *p* < 0.001. The *p*-values in animal experiments, for comparisons of more than two groups, the *LSD* test or *Tamhane* test in ANOVA was used. Mann-Whitney *U* test was used for non-normally distributed data. *ApoE⁻/⁻*, apolipoprotein E-deficient mice; Nx5/6, 5/6 nephrectomy; LDP, low dose probiotics; HDP, high dose probiotics; TG, triglycerides; TC, total cholesterol; LDL, low-density lipoprotein.


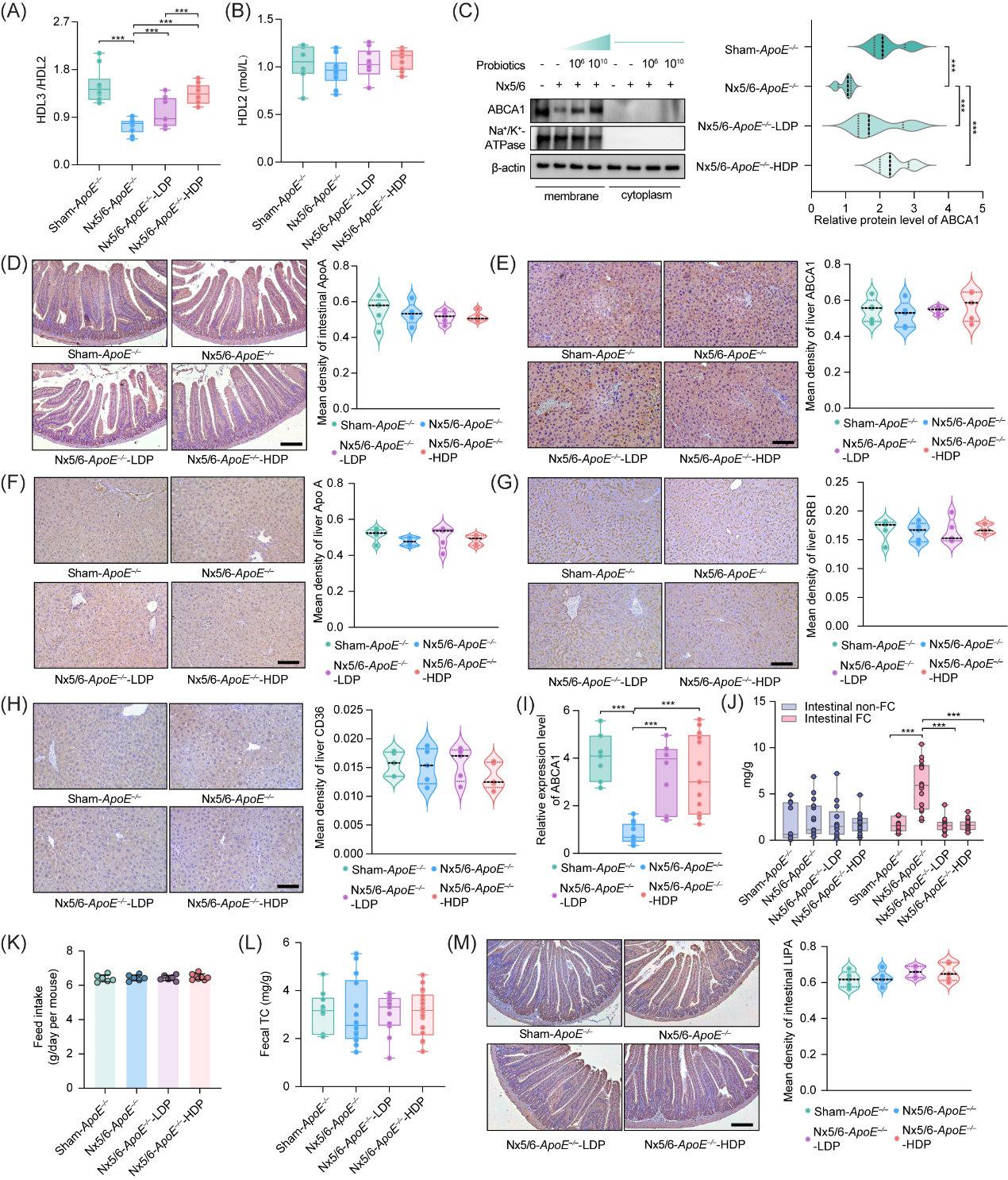


**Figure S6. Effect of probiotics on cholesterol metabolism in *ApoE^-/-^* mice with severe renal injury.** (A−B) Serum HDL3/HDL2 ratio and HDL3 concentration in mice after 3 months of probiotic gavage (*n =* 9). (C) Immunoblotting of small intestinal ABCA1 protein expression in four groups. Na+/K+-ATPase served as the membrane protein loading control; β-actin was used as the cytoplasmic protein control. Quantification of ABCA1 levels is shown on the right (*n =* 4). (D) Expression of ApoA in small intestine of mice after 3 months of probiotic gavage. Left: Immunofluorescent analysis of ApoA expression in the small intestine with four groups (*n =* 5), scale bars: 200 µm. Right: Quantification of staining levels in left. (E) Expression of ABCA1 in mouse liver after 3 months of probiotic gavage. Left: Immunohistochemistry analysis of ABCA1 expression in the liver with four groups (*n =* 5), scale bars: 100 µm. Right: Quantification of staining levels in left. (F) Expression of ApoA in mouse liver after 3 months of probiotic gavage. Left: Immunohistochemistry analysis of ApoA expression in the liver with four groups (*n =* 5), scale bars: 100 µm. Right: Quantification of staining levels in left. (G) Expression of SRBI in mouse liver after 3 months of probiotic gavage. Left: Immunohistochemistry analysis of SRBI expression in the liver with four groups, scale bars: 100 µm. Right: Quantification of staining levels in left (*n =* 5). (H) Expression of CD36 in mouse liver after 3 months of probiotic gavage. Left: Immunohistochemistry analysis of CD36 expression in the liver with four groups, scale bars: 100 µm. Right: Quantification of staining levels in left (*n =* 5). (I) Transcriptional analysis of small intestinal ABCA1 mRNA levels (Sham, *n =* 7; Nx5/6, *n =* 11; Nx5/6-LDP, *n =* 10; Nx5/6-HDP, *n =* 13). (J) Cholesterol distribution in the small intestine after 3 months of probiotic gavage (Sham, *n =* 10; Nx5/6, *n =* 15; Nx5/6-LDP, *n =* 13; Nx5/6-HDP, *n =* 18), (FC, Free cholesterol). (K) Changes in food intake of mice after 3 months of probiotic gavage (*n =* 6). (L) Concentrations of cholesterol content in the faeces of mice after 3 months of probiotic gavage (Sham, *n =* 10; Nx5/6, *n =* 14; Nx5/6-LDP, *n =* 13; Nx5/6-HDP, *n =* 18). (M) Expression of LIPA in the small intestine of mice after 3 months of probiotic gavage. Left: Immunohistochemistry analysis of LIPA expression in the small intestine with four groups (*n =* 5), scale bars: 200 µm. Right: Quantification of staining levels in left. Significant differences between groups are denoted by asterisks: *: *p* < 0.05; **: *p* < 0.01; ***: *p* < 0.001. The *p*-values in animal experiments, for comparisons of more than two groups, the *LSD* test or *Tamhane* test in ANOVA was used. Mann-Whitney *U* test was used for non-normally distributed data. *ApoE⁻/⁻*, apolipoprotein E-deficient mice; Nx5/6, 5/6 nephrectomy; LDP, low dose probiotics; HDP, high dose probiotics; ABCA1, ATP binding cassette subfamily A member 1; ApoA, apolipoprotein A; SRB Ⅰ, scavenger receptor type B1; CD36, cluster of differentiation 36; LIPA, lipase A; TC, total cholesterol.


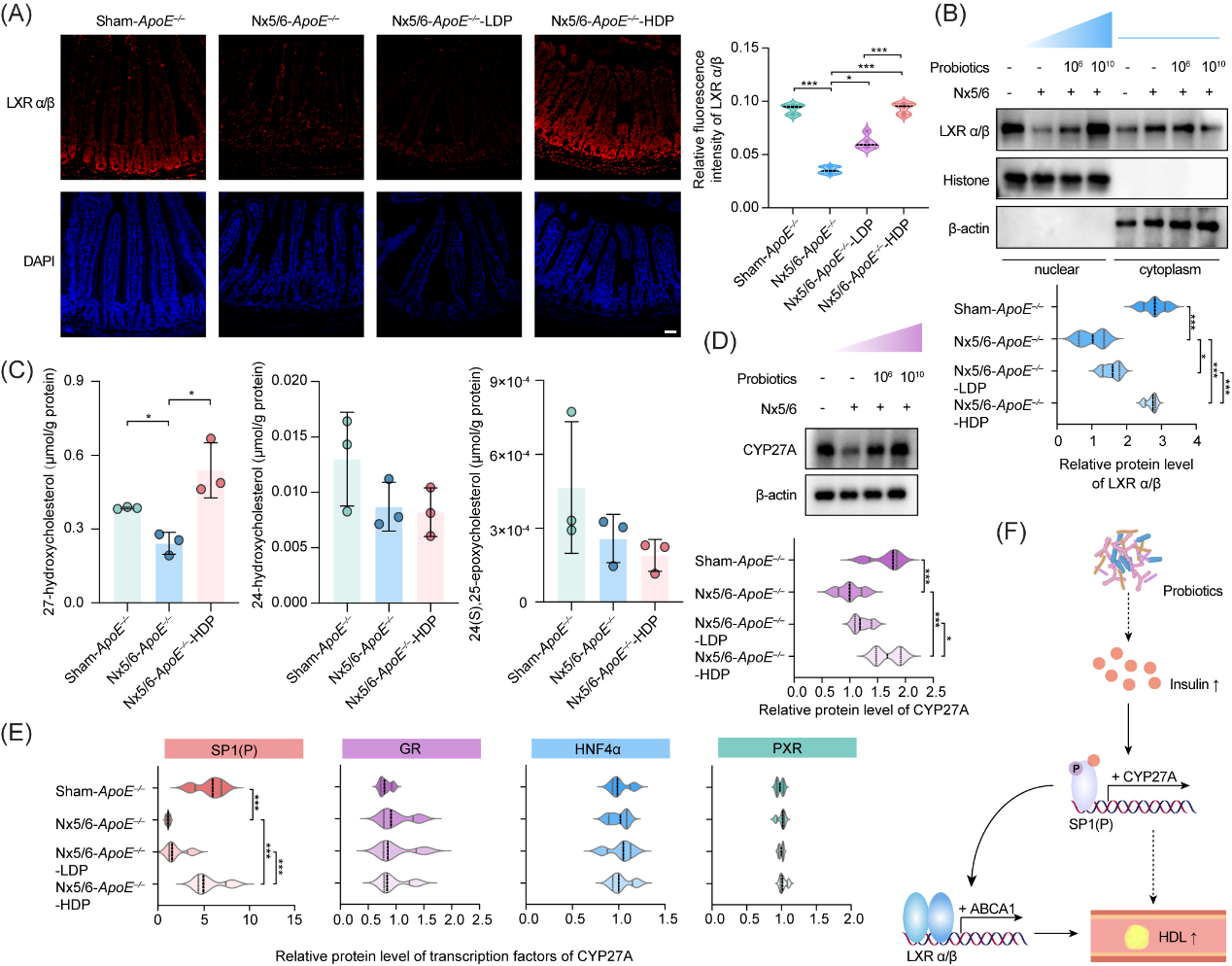


**Figure S7. Probiotics enhance small intestinal ABCA1 expression via the insulin-mediated SP1(P)-CYP27A-LXR α/β-ABCA1 pathway in nephropathic *ApoE^-/-^* mice.** (A-B) Activation of LXRα/β in the mouse small intestine after 3 months of probiotic gavage. (A) Immunofluorescent staining of LXRα/β expression in four groups (scale bar: 50 µm), with quantification of staining intensity (*n =* 5). (B) Nuclear LXRα/β protein levels analyzed by immunoblotting. Histone served as the nuclear protein loading control, and β-actin was the cytoplasmic control. Quantification of protein levels is shown (*n =* 4). (C) Concentrations of intestinal 27-hydroxycholesterol, 24-hydroxycholesterol and 24(S)25-epoxycholesterol in mice after 3 months of probiotic gavage (*n =* 3). (D) CYP27A protein levels in the small intestine analyzed by immunoblotting, with quantification of levels (*n =* 4). (E) Nuclear CYP27A transcription factor levels analyzed by immunoblotting, with histone as the nuclear protein loading control and β-actin as the cytoplasmic control. Quantification of levels is shown (*n =* 4). (F) Schematic summarizing the upregulation of small intestinal ABCA1 expression via the insulin-mediated SP1(P)-CYP27A-LXRα/β-ABCA1 pathway after probiotic intervention. Significant differences between groups are denoted by asterisks (*: *p* < 0.05; **: *p* < 0.01; ***: *p* < 0.001). The *p*-values in animal experiments, for comparisons of more than two groups, the *LSD* test or *Tamhane* test in ANOVA was used. Mann-Whitney *U* test was used for non-normally distributed data. *ApoE⁻/⁻*, Apolipoprotein E-deficient-deficient mice; Nx5/6, 5/6 nephrectomy; LDP, low dose probiotics; HDP, high dose probiotics; LXR α/β, liver X receptor α/β; CYP27A, cytochrome P450 family 27 subfamily A member 1; SP1(P), phosphorylated specificity protein 1; GR, glucocorticoid receptor; HNF4α, hepatocyte nuclear factor 4 α; PXR, pregnane X receptor; ABCA1, ATP binding cassette subfamily A member 1.


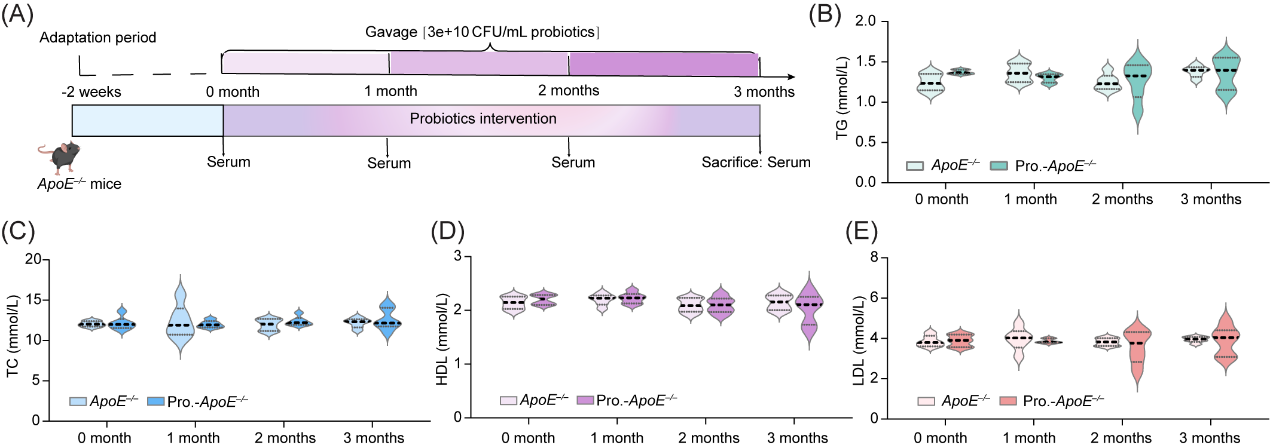


**Figure S8. Blood lipids Concentrations in *ApoE^-/-^* mice during 3 months of probiotic gavage (*n =* 6).** (A) Experimental design. (B−E) Concentrations of TC, TG, HDL, and LDL in mice during 3 months of gavage probiotics (*n =* 6). *ApoE⁻/⁻*, apolipoprotein E-deficient mice; TG, triglycerides; TC, total cholesterol; HDL, high-density lipoprotein; LDL, low-density lipoprotein.


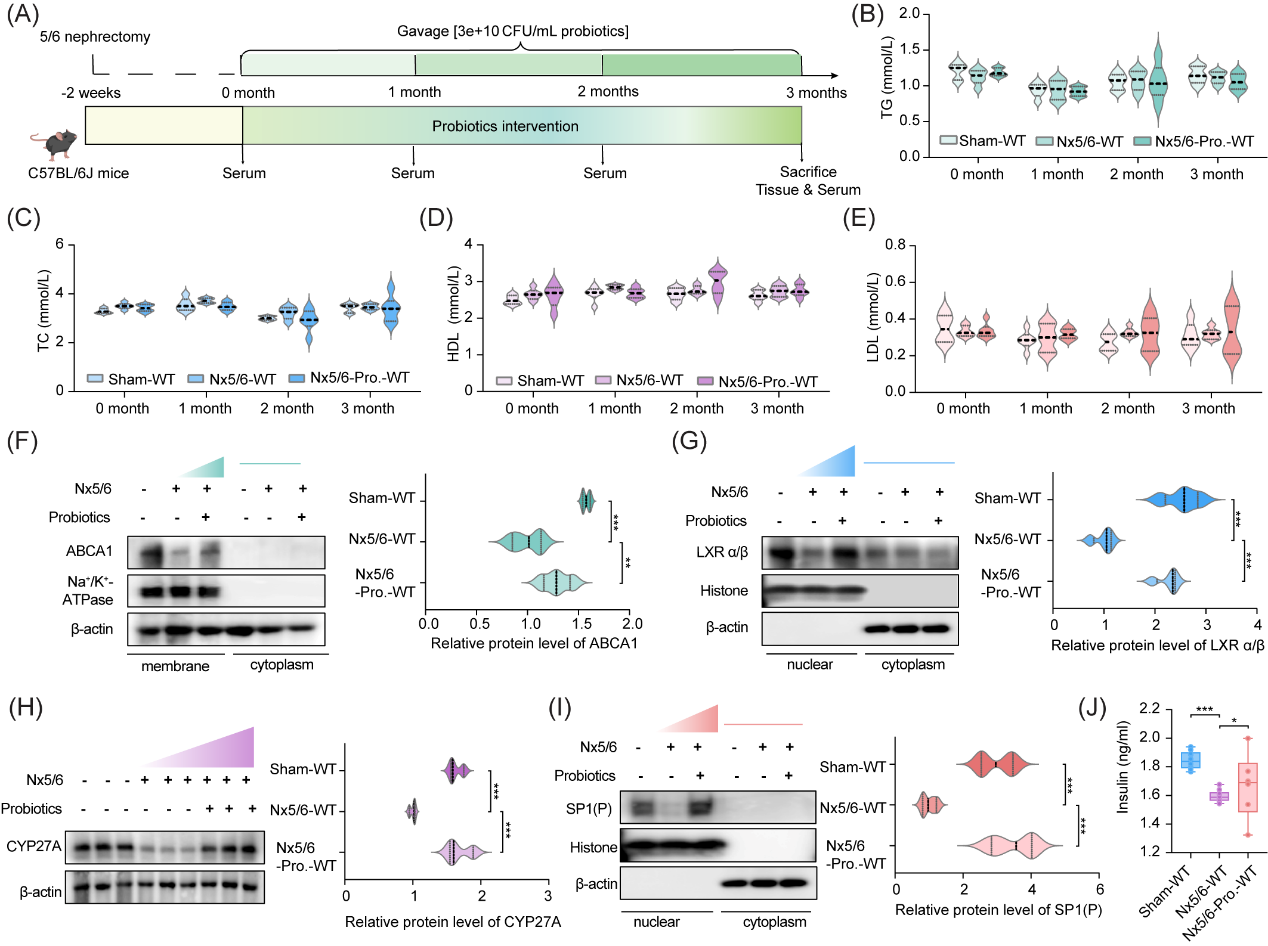


**Figure S9. Effect of probiotic supplementation on HDL content and synthesis in C57BL/6J mice.** (A) Experimental design. (B) Concentrations of TC, TG, HDL, and LDL in mice during 3 months of probiotic gavage (*n =* 6). (C) Expression of ABCA1 in mouse small intestine during 3 months of probiotic gavage. Left: Immunoblotting for the expression of ABCA1 protein in the small intestine with four groups. Na^+^/K^+^-ATPase was used as membrane protein loading control, whereas β-actin was cytoplasmic proteins control. Right: Quantification of protein levels in left (*n =* 4). (D) Activation of transcription factor LXR α/β of mouse small intestine ABCA1 during 3 months of probiotic gavage. Histone was used as nuclear protein loading control, whereas β-actin was cytoplasmic proteins control. Left: Immunoblotting for the expression of LXR α/β protein in the nucleus of small intestine with four groups. Right: Quantification of protein levels in left (*n =* 4). (E) Expression of CYP27A in the small intestine of mice after 3 months of probiotic gavage. Left: Immunoblotting for the expression of CYP27A protein in the small intestine with four groups. Right: Quantification of protein levels in left (*n =* 3). (F) Activation of CYP27A transcription factor SP1(P) in the small intestine of mice after 3 months of probiotic gavage. Left: Immunoblotting for the expression of SP1(P) protein in the nucleus of small intestine with four groups. Histone was used as nuclear protein loading control, whereas β-actin was cytoplasmic proteins control. Right: Quantification of protein levels in left (*n =* 4). (G) Insulin Concentrations in mice after 3 months of probiotic gavage. Asterisks indicated significant differences between the two groups: *: *p* < 0.05; **: *p* < 0.01; ***: *p* < 0.001. The *p*-values in animal experiments, for comparisons of more than two groups, the *LSD* test or *Tamhane* test in ANOVA was used. Mann-Whitney *U* test was used for non-normally distributed data. Nx5/6, 5/6 nephrectomy; WT, wild type mice; TG, triglycerides; TC, total cholesterol; HDL, high-density lipoprotein; LDL, low-density lipoprotein; ABCA1, ATP binding cassette subfamily A member 1; LXR α/β, liver X receptor α/β; CYP27A, cytochrome P450 family 27 subfamily A member 1; SP1(P), phosphorylated specificity protein 1.


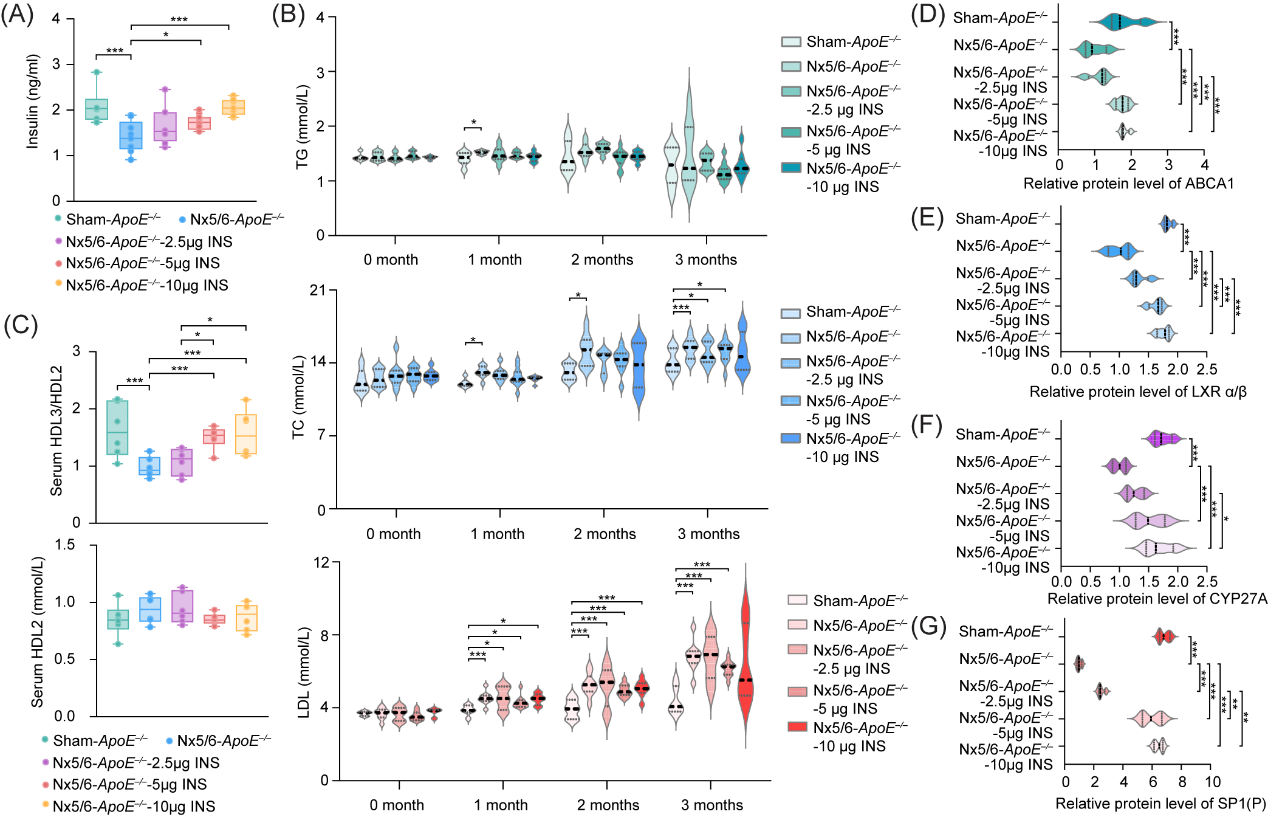


**Figure S10. Relation between insulin and HDL content in nephropathic *ApoE^-/-^* mice.** (A) Circulating insulin levels. (B) Serum levels of TG, TC and LDL after 3 months of insulin injection (*n =* 6). (C)Serum HDL3/HDL2 ratio and HDL3 concentration in mice after 3 months of insulin injection (*n =* 9). (D) Expression of small intestinal ABCA1 protein 3 months after insulin injection. (*n =* 4). (E) Activation of ABCA1 transcription factor LXRα/β in the small intestine after insulin injection. (*n =* 4). (F) CYP27A protein expression in the small intestine after insulin injection. (*n =* 4). (G) Activation of CYP27A transcription factor SP1(P) in the small intestine. (*n =* 4). Asterisks denote significant differences: *: *p* < 0.05; **: *p* < 0.01; ***: *p* < 0.001. The *p*-values in animal experiments, for comparisons of more than two groups, the *LSD* test or *Tamhane* test in ANOVA was used. Mann-Whitney *U* test was used for non-normally distributed data. *ApoE⁻/⁻*, apolipoprotein E-deficient mice; Nx5/6, 5/6 nephrectomy; INS, insulin; HDL, high-density lipoprotein; TG, triglycerides; TC, total cholesterol; LDL, low-density lipoprotein; ABCA1, ATP binding cassette subfamily A member 1; LXR α/β, liver X receptor α/β; CYP27A, cytochrome P450 family 27 subfamily A member 1; SP1(P), phosphorylated specificity protein 1.


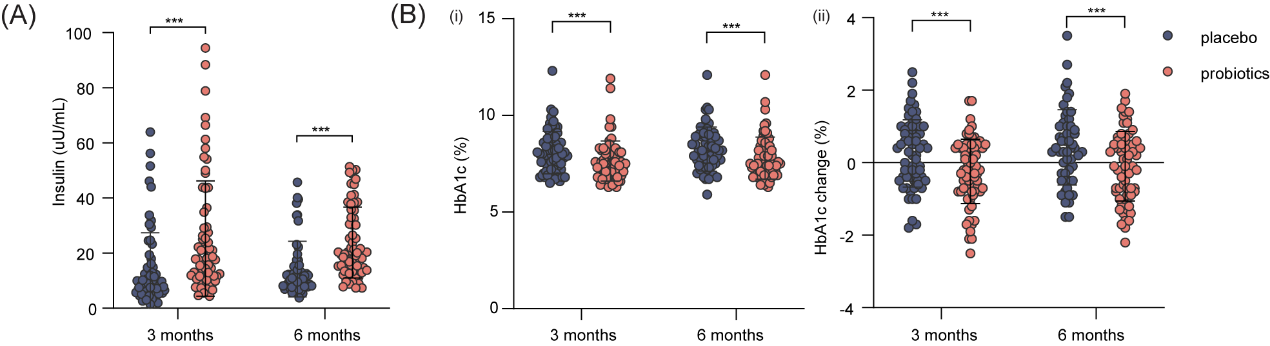


**Figure S11. Effect of probiotics on insulin and HbA1c in ESRD patients.** (A) Serum insulin levels in ESRD patients after 3 and 6 months of probiotics versus placebo. (B) Concentrations of HbA1c (i) and changes in HbA1c concentrations (ii) in ESRD patients taking probiotics compared to placebo for 3 months and 6 months. Sample sizes: probiotics group (3 months, *n =* 68; 6 months, *n =* 66), placebo group (3 months, *n =* 65; 6 months, *n =* 54). Significant differences: ***: *p* < 0.001 (Mann-Whitney *U* test). HbAc1c, glycated hemoglobin.


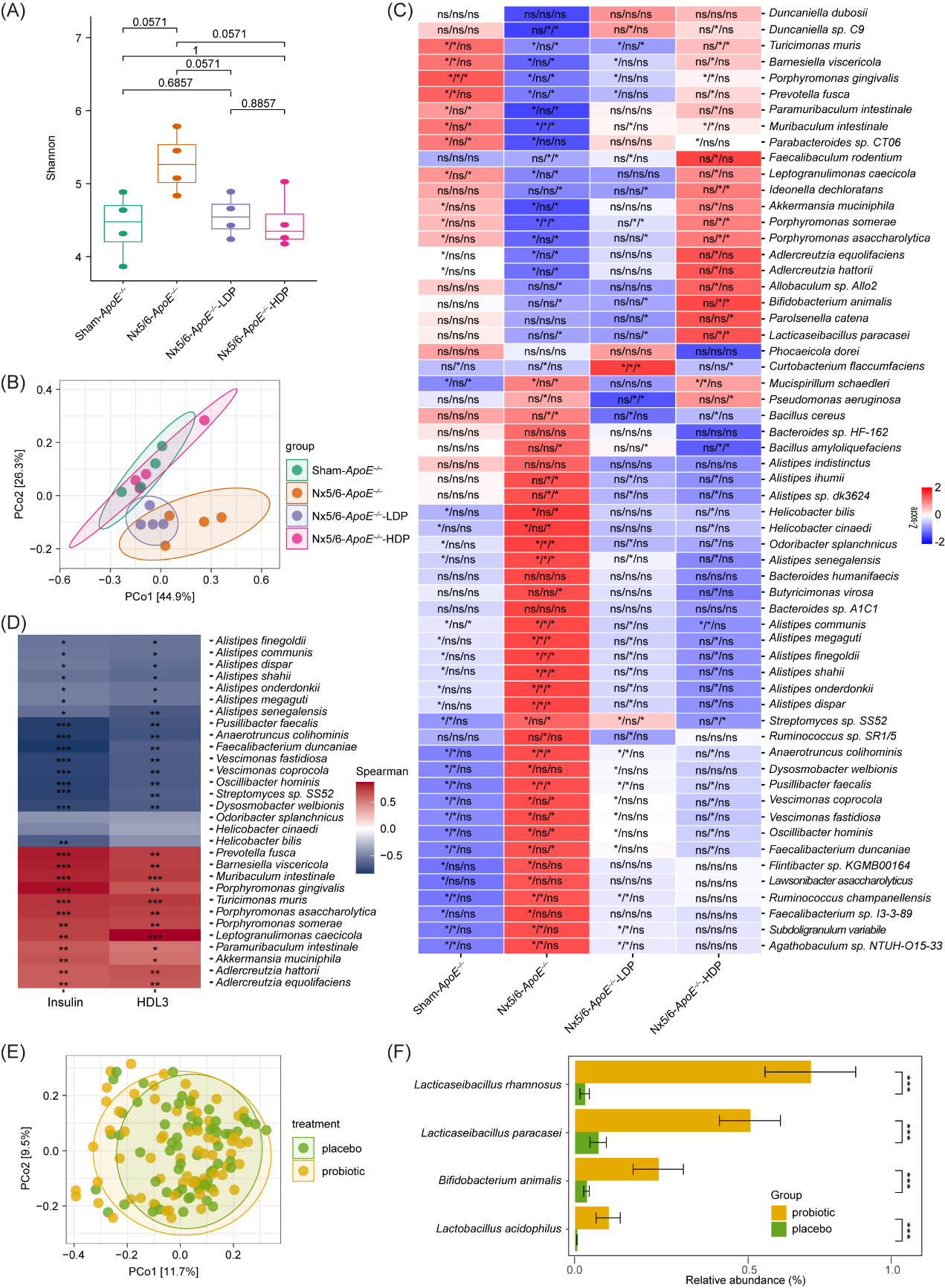


**Figure S12. Comparative analysis of the gut microbial composition in fecal samples from nephropathic** ***ApoE^-/-^* mice or patients with ESRD treated with probiotics.** (A-D) Gut microbial diversity in nephropathic *ApoE^-/-^* mice (*n =* 4). (A) Shannon diversity index of bacterial communities. (B) PCoA plot showing β diversity distribution among severe renal injury *ApoE^-/-^* mouse samples. (C) Changes in major differential species among groups of fecal metagenomes in *ApoE^-/-^* mice after probiotic treatment (*n =* 4). Heatmap shows mean relative abundance of species post-treatment, with significance calculated using two-sided Wilcoxon rank-sum tests (*: *p* < 0.05; ns: not significant). Non-significant results are omitted. (D) Correlation of the main differential species abundance and insulin and HDL3 levels in Sham-*ApoE^-/-^* and Nx5/6-*ApoE^-/-^* groups (*n =* 4). Heatmap shows Spearman correlation coefficients, with significance denoted as *: *p* < 0.05; **: *p* < 0.01; ***: *p* < 0.001. (E-F) Gut microbial diversity in ESRD patients pre- and post-probiotic treatment (*n =* 133). (E) PCoA plot showing β diversity distribution in ESRD patients from baseline to post-treatment. (F) Probiotic colonization in ESRD patients (*n =* 133). Heatmap shows Spearman correlation coefficients with significance denoted as *: *p* < 0.05; **: *p* < 0.01 (*n =* 133). *ApoE⁻/⁻*, apolipoprotein E-deficient mice; Nx5/6, 5/6 nephrectomy; LDP, low dose probiotics; HDP, high dose probiotics.

**Supplementary References**

1. Cheung, Alfred K., Mark J. Sarnak, Guofen Yan, Michael Berkoben, Robert Heyka, Allen Kaufman, Julia Lewis, et al. 2004. “Cardiac diseases in maintenance hemodialysis patients: results of the HEMO study.” *Kidney international* 65(6): 2380–2389. <https://doi.org/10.1111/j.1523-1755.2004.00657.x>

2. Stampfer, Meir J., Frank M. Sacks, Simonetta Salvini, Walter C. Willett, and Charles H. Hennekens. 1991. “A prospective study of cholesterol, apolipoproteins, and the risk of myocardial infarction.” *The New England journal of medicine* 325(6): 373–381. <https://doi.org/10.1056/NEJM199108083250601>

3. Sweetnam, Peter M., Colin H. Bolton, John W.G. Yarnell, David Bainton, Ian A. Baker, Peter C.Elwood, Norman E. Miller. 1994. “Associations of the HDL2 and HDL3 cholesterol subfractions with the development of ischemic heart disease in british men. The Caerphilly and Speedwell Collaborative Heart Disease Studies.” *Circulation* 90(2): 769–774. <https://doi.org/10.1161/01.cir.90.2.769>

4. Koch, M., B. Kutkuhn, E. Trenkwalder, D. Bach, B. Grabensee, H. Dieplinger, F. Kronenberg. 1997. “Apolipoprotein B, fibrinogen, HDL cholesterol, and apolipoprotein(a) phenotypes predict coronary artery disease in hemodialysis patients.” *Journal of the American Society of Nephrology : JASN* 8(12): 1889–1898. <https://doi.org/10.1681/ASN.V8121889>

5. Chiang, Chih-Kang Chiang, Tai-I Ho, Shih-Ping Hsu, Yu-Sen Peng, Mei-Fen Pai, Shao-Yu Yang, Kuan-Yu Hung, et al. 2005. “Low-density lipoprotein cholesterol: association with mortality and hospitalization in hemodialysis patients.” *Blood purification* 23(2): 134–140. <https://doi.org/10.1159/000083529>

6. Kilpatrick, Ryan D., Charles J. McAllister, Csaba P. Kovesdy, Stephen F. Derose, Joel D. Kopple, Kamyar Kalantar-Zadeh. 2007. “Association between serum lipids and survival in hemodialysis patients and impact of race.” *Journal of the American Society of Nephrology* 18(1): 293–303. <https://doi.org/10.1681/ASN.2006070795>

7. Krane, Vera, Karl Winkler, Christiane Drechsler, Jürgen Lilienthal, Winfried März, Christoph Wanner; German Diabetes and Dialysis Study Investigators. 2009. “Association of LDL cholesterol and inflammation with cardiovascular events and mortality in hemodialysis patients with type 2 diabetes mellitus.” *American journal of kidney diseases : the official journal of the National Kidney Foundation* 54(5): 902–911. <https://doi.org/10.1053/j.ajkd.2009.06.029>

8. Shoji, Tetsuo, Ikuto Masakane, Yuzo Watanabe, Kunitoshi Iseki, Yoshiharu Tsubakihara; Committee of Renal Data Registry, Japanese Society for Dialysis Therapy. 2011. “Elevated non-high-density lipoprotein cholesterol (non-HDL-C) predicts atherosclerotic cardiovascular events in hemodialysis patients.” *Clinical journal of the American Society of Nephrology : CJASN* 6(5): 1112–1120. <https://doi.org/10.2215/CJN.09961110>

9. Moradi, Hamid, Elani Streja, Moti L Kashyap, Nosratola D. Vaziri , Gregg C. Fonarow, Kamyar Kalantar-Zadeh. 2014. “Elevated high-density lipoprotein cholesterol and cardiovascular mortality in maintenance hemodialysis patients.” *Nephrology, dialysis, transplantation : official publication of the European Dialysis and Transplant Association - European Renal Association* 29(8): 1554–1562. <https://doi.org/10.1093/ndt/gfu022>

10. Peev, Vasil, Ali Nayer, Gabriel Contreras. 2014. “Dyslipidemia, malnutrition, inflammation, cardiovascular disease and mortality in chronic kidney disease.” *Current opinion in lipidology* 25(1): 54–60. <https://doi.org/10.1097/MOL.0000000000000045>

11. Kaysen, George A., Xiaoling Ye, Jochen G. Raimann, Yuedong Wang, Alice Topping, Len A Usvyat, Stefano Stuard, et al. 2018. “Lipid levels are inversely associated with infectious and all-cause mortality: international MONDO study results.” *Journal of lipid research* 59(8): 1519–1528. <https://doi.org/10.1194/jlr.P084277>

12. Lamprea-Montealegre, Julio Alejandro, Natalie Staplin, William G. Herrington, Richard Haynes, Jonathan Emberson, Colin Baigent, Ian H de Boer, et al. 2020. “Apolipoprotein B, triglyceride-rich lipoproteins, and risk of cardiovascular events in persons with CKD.” *Clinical journal of the American Society of Nephrology : CJASN* 15(1): 47–60. <https://doi.org/10.2215/CJN.07320619>

13. Ebert, T., A. R. Qureshi, C. Lamina, J. Fotheringham, M. Froissart, K-U Eckardt, D. C. Wheeler, et al. 2021. “Time-dependent lipid profile inversely associates with mortality in hemodialysis patients - independent of inflammation/malnutrition.” *Journal of internal medicine* 290(4): 910–921. <https://doi.org/10.1111/joim.13291>

14. Lee, Wen-Chin, Jin-Bor Chen, Sin-Hua Moi, Cheng-Hong Yang. 2021. “Association of proportion of the HDL-cholesterol subclasses HDL-2b and HDL-3 and macrovascular events among patients undergoing hemodialysis.” *Scientific reports* 11(1): 1871. <https://doi.org/10.1038/s41598-021-81636-3>

15. Wang, Xifan, Songtao Yang, Shenghui Li, Liang Zhao, Yanling Hao, Junjie Qin, Lian Zhang, et al. 2020. “Aberrant gut microbiota alters host metabolome and impacts renal failure in humans and rodents.” *Gut* 69(12): 2131–2142. <https://doi.org/10.1136/gutjnl-2019-319766>

16. Lam, Sin Man, Chao Zhang, Zehua Wang, Zhen Ni, Shaohua Zhang, Siyuan Yang, Xiahe Huang, et al. 2021. “A multi-omics investigation of the composition and function of extracellular vesicles along the temporal trajectory of COVID-19.” *Nature metabolism* 3(7): 909–922. <https://doi.org/10.1038/s42255-021-00425-4>

17. Zhang, Qi, Guang Li, Wen Zhao, Xifan Wang, Jingjing He, Limian Zhou, Xiaoxu Zhang, et al. 2024. “Efficacy of *Bifidobacterium animalis subsp. lactis BL-99* in the treatment of functional dyspepsia: a randomized placebo-controlled clinical trial.” *Nature communications* 15(1): 227. <https://doi.org/10.1038/s41467-023-44292-x>

18. Chen, Shifu, Yanqing Zhou, Yaru Chen, Jia Gu. 2018. “Fastp: an ultra-fast all-in-one FASTQ preprocessor.” *Bioinformatics (Oxford, England)* 34(17): i884–i890. <https://doi.org/10.1093/bioinformatics/bty560>

19. Langmead, Ben, Steven L Salzberg. 2012. “Fast gapped-read alignment with bowtie 2.” *Nature methods* 9(4): 357–359. <https://doi.org/10.1038/nmeth.1923>

20. Beghini, Francesco, Lauren J McIver, Aitor Blanco-Míguez, Leonard Dubois, Francesco Asnicar, Sagun Maharjan, Ana Mailyan, et al. 2021. “Integrating taxonomic, functional, and strain-level profiling of diverse microbial communities with bioBakery 3.” *Elife* 10, e65088. <https://doi.org/10.7554/eLife.65088>
